# Harmane induces apoptosis through RRM2B and suppresses colorectal cancer progression

**DOI:** 10.1128/msystems.01704-25

**Published:** 2026-06-09

**Authors:** Guifang Li, Li Li, Peipei Shen, Jifan Wang, Yanyan Feng, Yangmeng Yu, Hejia Xu, Hunan Wang, JiaXuan Li, XiangQian Zheng, Yong Mao

**Affiliations:** 1 Tianjin Key Laboratory of Digestive Cancer, Tianjin Medical University Cancer Institute and Hospital, National Clinical Research Center for Cancer, Tianjin's Clinical Research Center for Cancer, Tianjin, China; 2 Department of Oncology, The Affiliated Hospital of Jiangnan University, Wuxi, China; 3 Department of Oncology, Wuxi Eighth People’s Hospital, Wuxi, China; 4 Wuxi Medical College of Jiangnan University, Wuxi, China; 5 Department of Thyroid and Neck Cancer, Key Laboratory of Cancer Prevention and Therapy, Tianjin Medical University Cancer Institute and Hospital, National Clinical Research Center for Cancer, Tianjin's Clinical Research Center for Cancer, Tianjin, China; Chinese Academy of Sciences, Shanghai, China

**Keywords:** colorectal cancer, harmane, apoptosis, RRM2B, gut microbiota

## Abstract

Colorectal cancer (CRC) is a common malignant tumor of the digestive system, and chemotherapy resistance often leads to poor patient prognosis. Harmane, a natural indole alkaloid, is found in Leguminosae plants (particularly those of the Crotalaria genus), as well as in mammalian tissues and certain food products. It exhibits potential anticancer activities through multiple mechanisms in various cancers, including liver, breast, and thyroid cancers. However, the role of harmane in the treatment of CRC remains unclear. In this study, we demonstrate that harmane induces cell cycle arrest and apoptosis in CRC cells via the p53–RRM2B axis. Furthermore, at the level of the gut microbiota, harmane reshapes microbial composition, thereby contributing to its anti-tumor effects.

**IMPORTANCE:**

This study is the first to demonstrate a progressive decline of harmane levels in the gut from healthy individuals to advanced adenoma and CRC patients, suggesting its potential protective role in CRC development. We further found that harmane promotes CRC cell apoptosis via RRM2B-mediated regulation, revealing the underlying molecular mechanism. Moreover, *in vivo* experiments showed that harmane can modulate gut microbial composition and its derived metabolites, and fecal microbiota transplantation experiments indicated that harmane exerts anticancer effects by regulating both the gut microbiota and microbial metabolites. This study proposes a novel therapeutic strategy for CRC, highlighting the importance of incorporating gut microbiota modulation into cancer treatment.

## INTRODUCTION

Colorectal cancer (CRC) ranks among the most prevalent and lethal malignancies worldwide, characterized by high incidence and mortality rates (1). The current standard treatment strategy involves a multimodal approach combining surgical resection with adjuvant chemotherapy, radiotherapy, and targeted therapies. Despite these interventions, treatment resistance remains a major clinical challenge. Once resistance develops, the median survival time often drops to less than 12 months (2, 3). The colorectum hosts a complex and dynamic microbial ecosystem, and emerging evidence highlights the gut microbiome’s significant role in modulating CRC progression. Gut microbiota exerts either anti-tumor or pro-tumor effects primarily through its metabolic outputs. These metabolites originate from diverse sources: some are diet-derived, others are produced through human metabolic pathways, and many are synthesized or transformed by gut microbes themselves (4). Harmane, a naturally occurring indole alkaloid, is primarily found in certain plants and food products and can also be endogenously synthesized by human and microbial metabolic pathways, notably through tryptophan metabolism (5–7). Growing preclinical studies suggest that harmane possesses broad anticancer properties. For instance, it has been shown to inhibit neuroblastoma progression through mitochondrial-dependent apoptotic signaling mediated by oxidative stress (8). Novel derivatives such as B-9-3 exhibit enhanced anticancer activity by inducing apoptosis and inhibiting cell migration (9). Furthermore, harmane combined with β-cyclodextrin demonstrates synergistic effects in promoting apoptosis even in BRAF-mutant melanoma cells with acquired chemoresistance (10). Although these findings underscore the potential of harmane as an anticancer agent, its precise mechanisms in suppressing CRC cells, particularly its interplay with the gut microbiome, remain incompletely elucidated. Further investigation is needed to determine whether harmane alters the microbial community to enhance anti-tumor responses and the involved molecular mechanisms.

Ribonucleotide reductase regulatory subunit M2B (RRM2B), also known as p53R2, is a critical subunit of the ribonucleotide reductase (RNR) complex responsible for the *de novo* synthesis of deoxyribonucleotides (dNTPs), which are essential for DNA replication and repair (11). RRM2B is constitutively expressed and strongly induced under various stress conditions, including DNA damage, oxidative stress, and hypoxia (12, 13). It plays a vital role in maintaining mitochondrial DNA (mtDNA) stability, nucleotide pool balance, and cellular responses to genotoxic insults (14). On one hand, RRM2B is known to facilitate DNA damage repair and support cell survival by providing adequate dNTPs for nuclear and mitochondrial DNA maintenance (15). On the other hand, there is emerging evidence that RRM2B may participate in pro-apoptotic processes under certain conditions. For instance, caspase-dependent proteolysis of RRM2B has been observed during apoptosis, suggesting its potential role in amplifying cell death signals (16). Furthermore, silencing of RRM2B has been shown to enhance DNA damage-induced apoptosis in a p53-dependent manner in some cancer models, due to impaired DNA repair and growth arrest pathways (17). However, overexpression of RRM2B under other circumstances, particularly in response to certain therapeutic agents, may promote apoptotic pathways, though the precise mechanisms remain unclear. Given that RRM2B is transcriptionally regulated by p53 and may interface with mitochondrial apoptosis pathways, it is plausible that RRM2B serves as a downstream mediator or modulator of harmane-induced cytotoxicity (16). Nevertheless, the exact nature of this interplay, whether RRM2B potentiates or attenuates harmane’s pro-apoptotic effects, warrants further investigation. Therefore, elucidating the molecular mechanisms through which RRM2B influences apoptosis, particularly in the context of harmane treatment in CRC, may hold significant promise for developing new therapeutic strategies.

The gut microbiota plays a pivotal role in CRC development, treatment resistance, and metastatic progression, drawing significant scientific interest due to its profound influence on intestinal homeostasis and disease pathogenesis (18). Dysbiosis, characterized by an imbalance between beneficial and pathogenic bacteria, disrupts intestinal barrier integrity, modulates immune responses, and alters metabolic processes, thereby contributing critically to CRC initiation and progression. Pathogenic bacteria, such as polyketide synthase (PKS)-expressing *Escherichia coli*, promote carcinogenesis through genotoxic mechanisms, including the production of colibactin, which induces DNA double-strand breaks and chromosomal instability (19). *Fusobacterium nucleatum* facilitates tumor immune evasion by recruiting myeloid-derived suppressor cells, activating inflammatory pathways via TLR/MyD88 signaling, and enhancing chemoresistance and metastasis (20). Conversely, commercial probiotics like *Lactobacillus* and *Bifidobacterium* ferment dietary fibers to generate short-chain fatty acids, especially butyrate, which exerts anti-tumor effects by enhancing epithelial apoptosis, reinforcing mucosal barrier function, and modulating anti-inflammatory immune responses (21). Clinically, gut microbiota composition significantly influences treatment outcomes. Reduced microbial diversity and enrichment of specific taxa (such as *Firmicutes*) are associated with resistance to chemotherapy and immune checkpoint inhibitors. *F. nucleatum* is particularly related to dampened therapeutic efficacy and aggressive tumor phenotypes (22). Harmane may interact with the gut microbiome, potentially influencing microbial composition or metabolic activity, though its specific mechanisms remain underexplored. Investigating harmane’s impact on gut microbial ecology in murine CRC models will be essential for elucidating its role in microbiome-host crosstalk and assessing its translational potential in CRC therapy. Consequently, modulating the gut microbiome represents a promising adjunctive strategy for improving CRC treatment.

## MATERIALS AND METHODS

### Analysis of publicly available data sets

Metabolomics data and corresponding sample metadata were downloaded from the study in reference 23. Initial data quality assessment was performed to identify and exclude outliers and low-quality samples. Processing of raw peak data included noise reduction and feature filtering. Peak detection, retention time correction, and alignment were performed using XCMS. The resulting data were *z*-score normalized to reduce technical variance and improve cross-sample comparability. Differential abundance analysis was conducted using Student’s *t*-test or one-way analysis of variance (ANOVA) with false discovery rate (FDR) correction for multiple comparisons. Metabolites with an FDR-adjusted *P*-value < 0.05 and |log₂ fold change| > 1 were considered statistically significant. Significant metabolites were mapped to biochemical pathways using KEGG for functional enrichment analysis.

### Cell lines and culture

The human CRC cell lines SW480 and HCT8 were obtained from the American Type Culture Collection (ATCC, USA) in 2015. The mouse-derived CRC cell line MC38 was purchased from the Cell Bank of the Chinese Academy of Sciences (Shanghai, China) in 2016. SW480 and HCT8 cells were cultured in DMEM medium (Gibco, USA) supplemented with 10% fetal bovine serum (Gibco, USA), 100 U/mL penicillin, and 100 μg/mL streptomycin. MC38 cells were cultured in RPMI-1640 medium (Gibco, USA) containing 10% FBS and the same concentration of antibiotics. All cell lines were incubated at 37°C in a humidified atmosphere of 5% CO_2_.

### CCK-8, colony formation, and EdU incorporation assay

For CCK-8 assay, cells were seeded at a density of 4 × 10^3^ cells per well in 96-well plates and allowed to adhere overnight. Each experimental condition was performed in triplicate. Following overnight incubation, CRC cells were treated with either 100 μM harmane (MedChemExpress, USA) or a vehicle control for 24 h. After treatment, 10 μL of CCK-8 solution (Vazyme, China) was added directly to each well, and the plates were incubated at 37°C for 4 h. Cell viability was determined at OD at 450 nm using a microplate reader (Thermo Scientific, USA).

For colony formation assay, CRC cells were seeded in 6-well plates at a low density of 500 cells per well and allowed to adhere overnight. Subsequently, the cells were exposed to 100 μM harmane or vehicle control for 24 h. After treatment, the medium was replaced with fresh complete medium, and the medium was replaced every 3 days. The cells were incubated for approximately 14 days. Colonies were fixed with 4% paraformaldehyde (PFA) and then stained with 0.5% (wt/vol) crystal violet solution. The colonies were imaged and counted manually.

For EdU assay, the cells were seeded in 96-well plates at 4 × 10^3^ cells per well and allowed to adhere overnight. CRC cells were treated with or without 100 μM harmane for 24 h. Cell proliferation was detected using the EdU-Apollo DNA proliferation assay kit (RiboBio, China) following the manufacturer’s instructions. Cell nuclei were counterstained with Hoechst 33,342. The images were captured using a fluorescence microscope (Nikon, Japan). The percentage of EdU-positive cells was calculated by dividing the number of EdU-positive nuclei by the total number of Hoechst-stained nuclei.

### Cell migration and invasion assay

Cell migration capacity was assessed using 8.0 μm transwell chambers (Corning, USA). Briefly, 5 × 10^3^ cells resuspended in 200 μL serum-free medium were seeded into the upper chamber. The lower chamber was filled with medium containing 10% FBS. After 24 h of incubation, non-migrated cells on the upper membrane surface were removed mechanically using a cotton swab. Cells that migrated to the lower surface were fixed with 4% PFA for 10 min, washed three times with PBS, and stained with 0.5% crystal violet solution for 5 min. Excess dye was removed by washing with PBS. Migrated cells on the lower membrane surface were visualized under an optical microscope (Nikon, Japan) and quantified from multiple random fields. All experimental conditions were identical except for the addition of harmane or the knockdown of RRM2B.

For cell invasion assay, transwell inserts pre-coated with Matrigel (Corning, USA) were used. Cells were seeded as described for the migration assay but incubated for 48 h, and the remaining steps were identical to those in the migration assay.

### Cell cycle analysis

CRC cells were seeded at a density of 8 × 10^3^ cells per well in 6-well plates and allowed to adhere overnight. Subsequently, the cells were treated with either 100 μM harmane or vehicle control for 24 h. After treatment, cells were fixed in 75% ethanol overnight at 4°C, treated with RNase A at 37 °C for 30 min, and stained with propidium iodide (PI) in PBS in the dark at room temperature for 30 min. PI fluorescence was measured using flow cytometry to establish the distribution of cells in the various phases of the cell cycle. FlowJo 8.1 software was used to perform the data analysis and interpretation.

### Analysis of apoptosis

CRC cells were seeded at a density of 8 × 10^3^ cells per well in 6-well plates and allowed to adhere overnight. Subsequently, the cells were treated with either 100 μM harmane or vehicle control for 24 h. After treatment, cells were washed twice with pre-cooled PBS and then resuspended in 100 μL binding buffer. Next, 5 μL of annexin V-FITC and 5 μL of PI were added, followed by incubation at room temperature in the dark for 15 min. Finally, 400 μL of binding buffer was added and mixed thoroughly. Samples were detected and analyzed using a BD FACSAria III flow cytometer.

### RNA sequencing (RNA-seq) and transcriptome analysis

HCT8 cells were treated with or without 100 µM harmane for 24 h and then washed twice with pre-cooled PBS. Total RNA was extracted using TRIzol reagent (Invitrogen), and RNA concentration and quality were assessed with a NanoDrop spectrophotometer and an Agilent 2100 Bioanalyzer. Only samples with an RNA Integrity number (RIN) ≥7.0 were used for subsequent library construction. All qualified samples were sent to Novogene for sequencing. Sequencing libraries were prepared using the NEBNext Ultra II RNA Library Prep Kit (New England Biolabs, USA) and sequenced on an Illumina NovaSeq 6000 platform to generate 150 bp paired-end reads. Raw data were quality-controlled using fastp, and clean reads were aligned to the human reference genome with the STAR aligner. Gene expression levels were quantified using featurecounts, and differential expression gene (DEG) analysis was performed with DESeq2. Genes with |log2FC| ≥ 1 and FDR < 0.05 were considered significant. Functional enrichment analyses of DEGs were carried out using clusterProfiler for GO and KEGG pathways analysis. The sequencing data generated in this study have been deposited in the NCBI Sequence Read Archive under BioProject accession number PRJNA1313752.

### Western blot analysis

Cells were washed twice with pre-cold PBS and lysed using RIPA buffer (Beyotime, China) supplemented with 1% protease and phosphatase inhibitors (Beyotime, China). The lysates were incubated on ice for 30 min and then centrifuged at 12,000 × *g* for 15 min at 4°C. The supernatant was collected, and protein concentration was determined using a BCA assay kit (Thermo Scientific, USA). Protein lysates were separated by 10% SDS-PAGE and transferred onto polyvinylidene fluoride (PVDF) membranes (Merck, USA). Membranes were blocked with 5% BSA (Absin, China) at room temperature for 1 h and then incubated overnight at 4°C with primary antibodies diluted in 5% BSA with gentle shaking. After incubation, membranes were washed three times with TBST and probed with HRP-conjugated secondary antibodies at room temperature for 1 h. Protein bands were visualized with the SuperSignal West Pico chemiluminescent substrate (Thermo Scientific, USA) and captured using an imaging system (Tanon, China). Band intensities were quantified using ImageJ software (NIH, USA). For each target protein, values were normalized to the corresponding GAPDH signal to correct for variations in protein loading. All primary and secondary antibodies used for this experiment are listed in Table S1.

### Small interfering RNA (siRNA) transfection

Cells were seeded in 6-well plates and incubated until they reached 80% confluence. Transfection complexes containing 50 nM of siRNA and Lipofectamine 3000 reagent (Invitrogen, USA) were added dropwise to the cells and incubated for 48 h. Three specific siRNA sequences targeting human RRM2B were listed as follows: si-RRM2B#1, AACAAUGAUCUCCCUGACCTT; si-RRM2B#2, UUUACAAUUCCAUCACUGGTT; and si-RRM2B#3, AAGACCUCUCUUCUUUAGCTT. A non-targeting scrambled siRNA (GenePharma, China) was used as a negative control.

### Quantitative real-time PCR

Total RNA was extracted from cells using TRIzol reagent according to the manufacturer’s protocol. Reverse transcription was performed with 1,000 ng of total RNA per sample using the PrimeScript RT Kit (Yeasen, China). Real-time qRT-PCR was conducted using SYBR Green Master Mix (Yeasen, China) on a CFX-96 real-time PCR Detection System (Bio-Rad, USA). The primer sequences used for target genes and the internal control (GAPDH) are shown in Table S2. Gene expression levels were normalized to GAPDH, and relative quantification was determined using the 2^-ΔΔCt^ method.

### Plasmid construction and cell transfection

Based on previously published studies, site-directed mutagenesis of RRM2B was performed (24, 25). The mutant plasmids were synthesized by Nanjing Corues Biotechnology (Nanjing, China). Transfection was carried out using Lipofectamine 3000 (Invitrogen) according to the manufacturer’s instructions. Transfection efficiency was verified by immunoblotting.

### Animal experiments

Male C57BL/6J mice (5–6 weeks old) were provided by Jiangsu Jice Biotechnology Co., Ltd. All mice were housed under specific pathogen-free conditions in a temperature-controlled environment (24 ± 2°C) with a 12-h light/dark cycle. To establish the tumor model, MC38 cells (5 × 10^5^ cells in 100 μL PBS) were subcutaneously inoculated into the flank of each mouse on day −7. When the tumor volume reached approximately 100 mm³ (day 0), the mice were randomly divided into three groups (*n* = 6 per group): control (PBS), harmane (30 mg/kg), and harmane (60 mg/kg). Harmane was administered by oral gavage (100 μL per mouse) every other day for 14 days (days 0, 2, 4, 6, 8, 10, 12, and 14). Body weight and tumor volume were recorded every 2 days. Tumor volume (*V*) was calculated using the formula *V* = (length × width²)/2. Fecal samples were collected at baseline (before the first administration) and at the endpoint (day 14) for subsequent gut microbiota and metabolomic analyses. On day 14, all mice were euthanized. Blood samples were collected to obtain serum for metabolomic profiling. Tumors were carefully excised, weighed, and divided into two parts for immunohistochemical analysis and further metabolic profiling. Additionally, cecal contents were harvested for subsequent metabolic and microbial community profiling.

For the fecal microbiota transplantation (FMT) experiment, mice were first subjected to antibiotic pretreatment to deplete the gut microbiota. Briefly, mice were orally administered a broad-spectrum antibiotic cocktail (ABX) once daily for seven consecutive days. The antibiotic mixture consisted of vancomycin (100 mg/kg), neomycin sulfate (200 mg/kg), metronidazole (200 mg/kg), and ampicillin (200 mg/kg). After antibiotic treatment, mice were randomly divided into two groups (*n* = 5 per group): the FMT-Ctrl group and the FMT-harmane group. Fecal samples were collected from the Ctrl and harmane groups in the previous experiment. One gram of feces was suspended in 5 mL of PBS. The suspension was then filtered through a 0.25 mm stainless steel mesh and centrifuged at 1,000 rpm for 5 min at 4 °C. The supernatant was collected for subsequent use. Mice in the two FMT groups were orally gavaged with 200 μL of fecal supernatant derived from the Ctrl or harmane donors every other day (26). Following antibiotic clearance, FMT was performed for five consecutive days to facilitate microbiota colonization. After stabilization of the gut microbiota, MC38 cells (5 × 10⁵ cells in 100 μL PBS) were subcutaneously inoculated into the mice. FMT was then continued until the end of the experiment.

### Hematoxylin and eosin (H&E) staining

Mouse tumor tissues were fixed in 4% formaldehyde for 48 h at room temperature, stored in ethanol, and embedded in paraffin. Tissue blocks were sectioned into 4 μm slices and stained with H&E reagent (BASO, China). Slides were observed under a light microscope (Nikon, Japan). Histopathological assessment was performed by two independent certified pathologists blinded to the treatment groups.

### Immunohistochemistry (IHC)

Paraffin-embedded mouse colorectal tumor tissues were sectioned at a thickness of 4 μm. Following deparaffinization in xylene and rehydration through a graded ethanol series, antigen retrieval was performed using ethylenediaminetetraacetic acid (EDTA)-sodium citrate buffer under microwave heating. After cooling to room temperature, sections were incubated with 3% hydrogen peroxide (H_2_O_2_) for 10 min to quench endogenous peroxidase activity. The sections were then incubated overnight at 4°C with primary antibodies listed in Table S1, followed by incubation with horseradish peroxidase (HRP)-conjugated anti-rabbit IgG polymer (Absin, China) for 1 h. Immunoreactivity was visualized using a 3,3’-diaminobenzidine (DAB) substrate kit (Absin, China). All stained sections were imaged using a microscope (Nikon, Japan). Quantitative analysis of staining intensity was performed with ImageJ software (NIH, USA).

### Untargeted metabolomics analysis

Fecal, serum, or tissue samples were collected and immediately stored at −80 °C until further analysis. Prior to extraction, samples were thawed and homogenized. Metabolites were extracted using a pre-chilled organic solvent mixture to precipitate proteins. After centrifugation, supernatants were dried, reconstituted in the mobile phase, and filtered through a 0.22 μm membrane filter.

Metabolite separation was performed using liquid chromatography-tandem mass spectrometry (LC-MS/MS) with a reversed-phase C18 column in both positive and negative ion modes. Raw data were processed using software such as XCMS or Progenesis QI for peak detection, alignment, and normalization. Metabolite annotation was performed using HMDB, METLIN, and KEGG databases. Multivariate statistical analyses, including principal component analysis (PCA) and partial least squares-discriminant analysis (PLS-DA), were applied to visualize group separation and identify significantly altered metabolites. Differential metabolites were selected based on fold change, variable importance in projection scores, and *P*-values.

### Metagenomic sequencing analysis

Fecal and cecal content samples were collected aseptically from mice and immediately stored at −80°C until further processing. Total microbial genomic DNA was extracted using the QIAamp Fast DNA Stool Mini Kit (Qiagen, Germany) following the manufacturer’s protocol. DNA quality and concentration were assessed with a NanoDrop spectrophotometer (Thermo Scientific, USA), and the integrity was verified via 0.8% agarose gel electrophoresis.

Sequencing libraries were constructed using the NEBNext Ultra DNA Library Prep Kit (New England Biolabs, USA). The libraries were quantified with a Qubit 2.0 Fluorometer (Invitrogen, USA), and the insert size was evaluated using an Agilent 2100 Bioanalyzer (Agilent Technologies, USA). Qualified libraries were subjected to paired-end sequencing (2 × 150 bp) on the Illumina NovaSeq 6000 platform (Illumina, USA). Raw reads were quality filtered using fastp, and host-derived sequences were removed by mapping to the mouse reference genome (GRCm38) with Bowtie2. High-quality clean reads were assembled *de novo* with MEGAHIT. Open reading frames were predicted using Prokka or MetaGeneMark, and non-redundant gene catalogs were constructed with CD-HIT. Taxonomic annotation was carried out against the NCBI non-redundant database using DIAMOND, while functional annotation was performed with the KEGG, eggNOG, and CAZy databases.

### Statistical analysis

All quantitative data are expressed as means ± standard error of the mean (SEM). Depending on the experimental design and number of groups, two-way ANOVA was used for multiple comparisons among two or more subgroups; one-way ANOVA was applied for comparisons among more than two groups; and an unpaired Student’s *t*-test was performed for comparisons between two groups. All *in vitro* experiments were performed in triplicate. Statistical analysis was performed using GraphPad Prism 9.0 (GraphPad Software, La Jolla, CA, USA). Differences with *P*-values < 0.05 were considered statistically significant.

## RESULTS

### Fecal metabolite profiles differ across healthy populations, patients with advanced adenoma, and CRC patients

**Fig 1:**
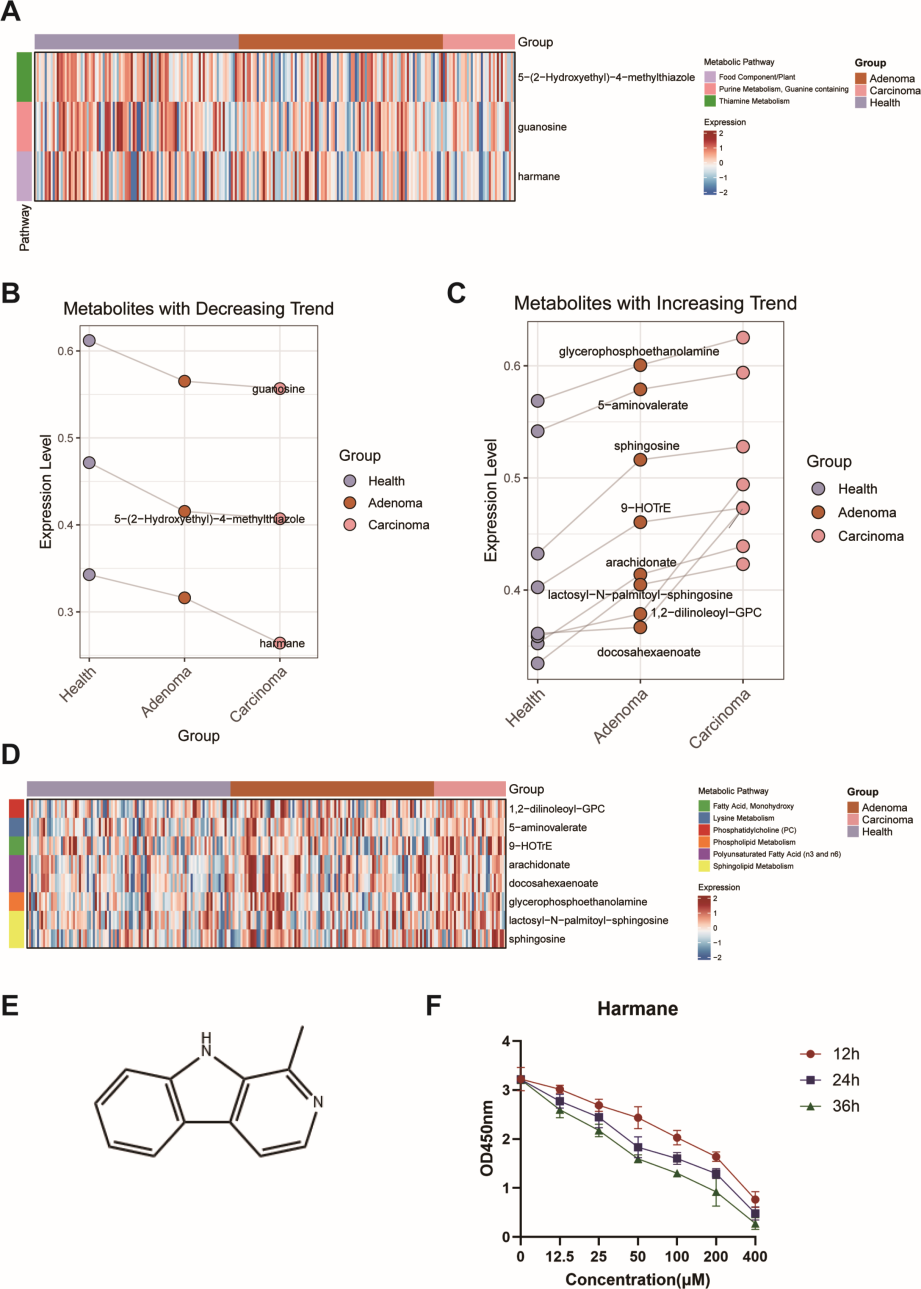
Dynamic remodeling of the gut metabolome across healthy populations, patients with advanced adenoma, and CRC patients. (**A**) Heatmap showing the KEGG pathway enrichment of gut metabolites that gradually decreased from healthy individuals to advanced adenoma patients and further to CRC patients. (**B**) Three metabolites showing consistent downward concentration trends along the disease continuum. (**C**) Eight metabolites demonstrating gradually increasing concentrations from healthy states to CRC states. (**D**) Heatmap illustrating the KEGG pathway enrichment of metabolites with increasing abundances. (**E**) Chemical structure of harmane. (**F**) Cytotoxic effect of harmane on CRC cells evaluated by CCK-8 assay.

We downloaded and analyzed the metabolomic profiles of fecal samples from healthy controls (*n* = 102), individuals with advanced adenoma (*n* = 102), and CRC patients (*n* = 36) (23). Significant alterations in metabolite levels were observed across the disease continuum. Specifically, the levels of 5-(2-hydroxyethyl)-4-methylthiazole, guanosine, and harmane showed a gradual decrease from healthy controls to patients with advanced adenoma and further to CRC patients (Fig. 1A and B). Functional enrichment analysis via the KEGG pathway platform indicated that these metabolites were associated with thiamine metabolism, purine metabolism, and food component-related pathways (Fig. 1B). Conversely, a distinct group of metabolites demonstrated progressively increasing abundances along the same disease spectrum. These included 1,2-dilinoleoyl-GPC (18:2/18:2), 5-aminovalerate, 9-HOTrE, arachidonate (20:4n6), docosahexaenoate (DHA; 22:6n3), glycerophosphoethanolamine, lactosyl-N-palmitoyl-sphingosine (d18:1/16:0), and sphingosine (Fig. 1C). The KEGG analysis revealed significant enrichment of these metabolites in fatty acid metabolism, monohydroxy metabolism, lysine metabolism, phosphatidylcholine biosynthesis, phospholipid metabolism, polyunsaturated fatty acid metabolism, and sphingolipid metabolism (Fig. 1D). These findings indicate substantial remodeling of the gut metabolome, encompassing microbial, dietary, and host-synthesized metabolites, throughout colorectal carcinogenesis. Metabolites showing progressive increases may potentially drive or accelerate malignant progression, whereas those exhibiting gradual decreases may possess tumor-suppressive properties. Based on these observations, we focused on subsequent functional validation on the metabolites that showed decreasing abundance patterns. *In vitro* cytotoxicity assays conducted in CRC cell lines demonstrated that harmane exerted potent concentration-dependent anticancer effects across various time points (Fig. 1E and F). In contrast, 5-(2-hydroxyethyl)-4-methylthiazole and guanosine did not show significant cytotoxic activity against CRC cells (Fig. S1A and B). Collectively, these results underscore the close association between gut metabolite dynamics and CRC pathogenesis. Certain metabolites, particularly harmane, may represent promising candidates for further investigation as potential therapeutic agents or biomarkers in CRC.

### Harmane inhibits proliferation, migration, and invasion of CRC cells and induces apoptosis

**Fig 2:**
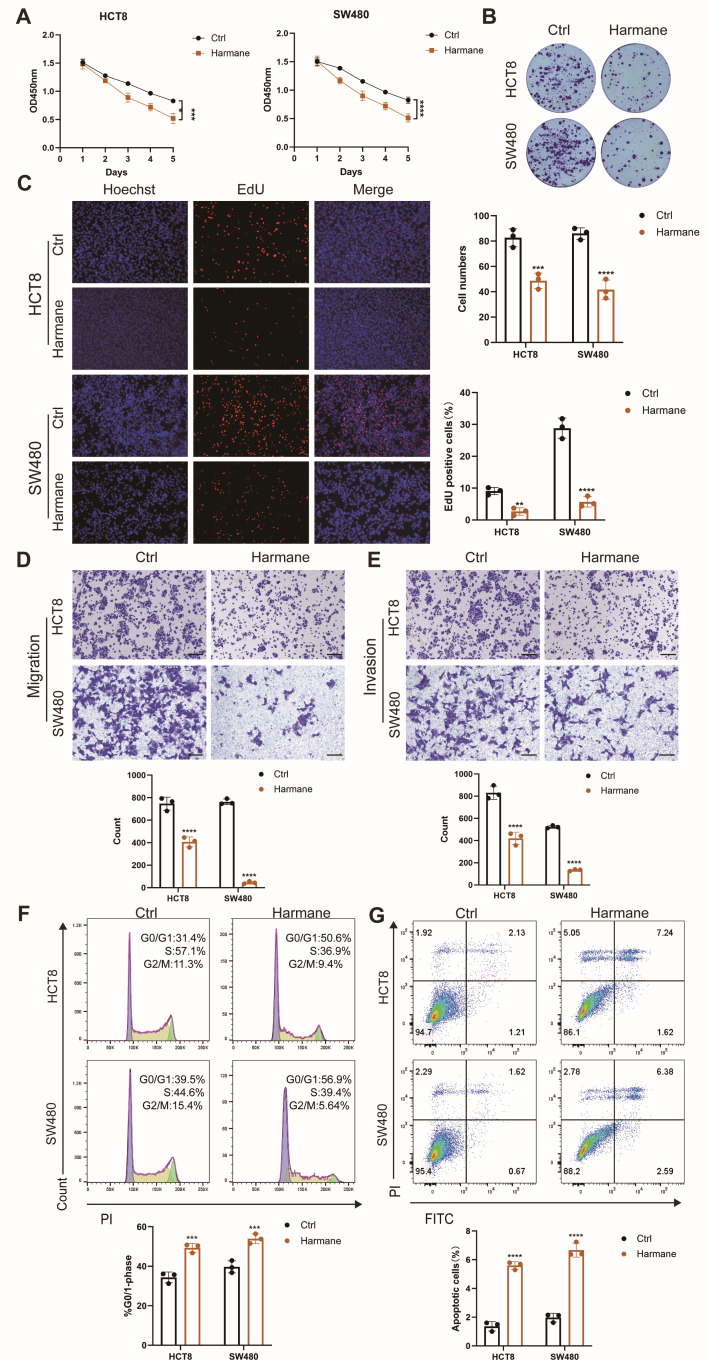
Harmane suppresses proliferation, migration, and invasion and induces apoptosis in CRC cells. (**A**) Viability of HCT8 and SW480 cells treated with 100 μM harmane for 24 h determined by CCK-8 assay. (**B, C**) Proliferative capacity of CRC cells evaluated by (**B**) colony formation assay and (**C**) EdU incorporation assay after 24 h harmane treatment. Scale bar: 100 μm. (**D, E**) Migratory and invasive abilities of CRC cells assessed by (**D**) transwell migration assay and (**E**) Matrigel invasion assay following 24 h harmane treatment. Scale bar: 100 μm. (**F**) Cell cycle distribution analyzed by flow cytometry using PI staining after 24 h harmane treatment. (**G**) Induction of apoptosis measured by annexin V/PI staining and flow cytometry. All data were derived from one representative experiment out of three independent biological replicates. Results are presented as means ± SEM. Statistical significance was determined using two-way ANOVA with Sidak’s multiple comparisons test. ***P < 0.005, ****P < 0.001.

Based on preliminary concentration screening, harmane was administered at a concentration of 100 μM to 2 CRC cell lines, HCT8 and SW480, for 24 h to assess its effects on cellular phenotypes. The CCK-8 assay showed that harmane significantly reduced CRC cell viability (Fig. 2A). Furthermore, colony formation and EdU assays demonstrated a substantial suppression of proliferative capacity in harmane-treated cells (Fig. 2B and C). Harmane also markedly impaired the metastatic potential of CRC cells, as evidenced by reduced migratory and invasive abilities in transwell migration and Matrigel invasion assays, respectively (Fig. 2D and E). Cell cycle analysis revealed that harmane-induced cell cycle arrest predominantly in the G0/G1 phase (Fig. 2F). Additionally, harmane treatment significantly increased the proportion of apoptotic CRC cells (Fig. 2G). These results indicate that harmane exerts antitumor effects in CRC cells by suppressing proliferation, migration, and invasion, as well as promoting apoptosis through induction of cell cycle arrest, highlighting its biological potential as a therapeutic agent to inhibit CRC progression.

### Harmane alters transcriptomic profiles and inhibits proliferation-related pathways in CRC cells

**Fig 3:**
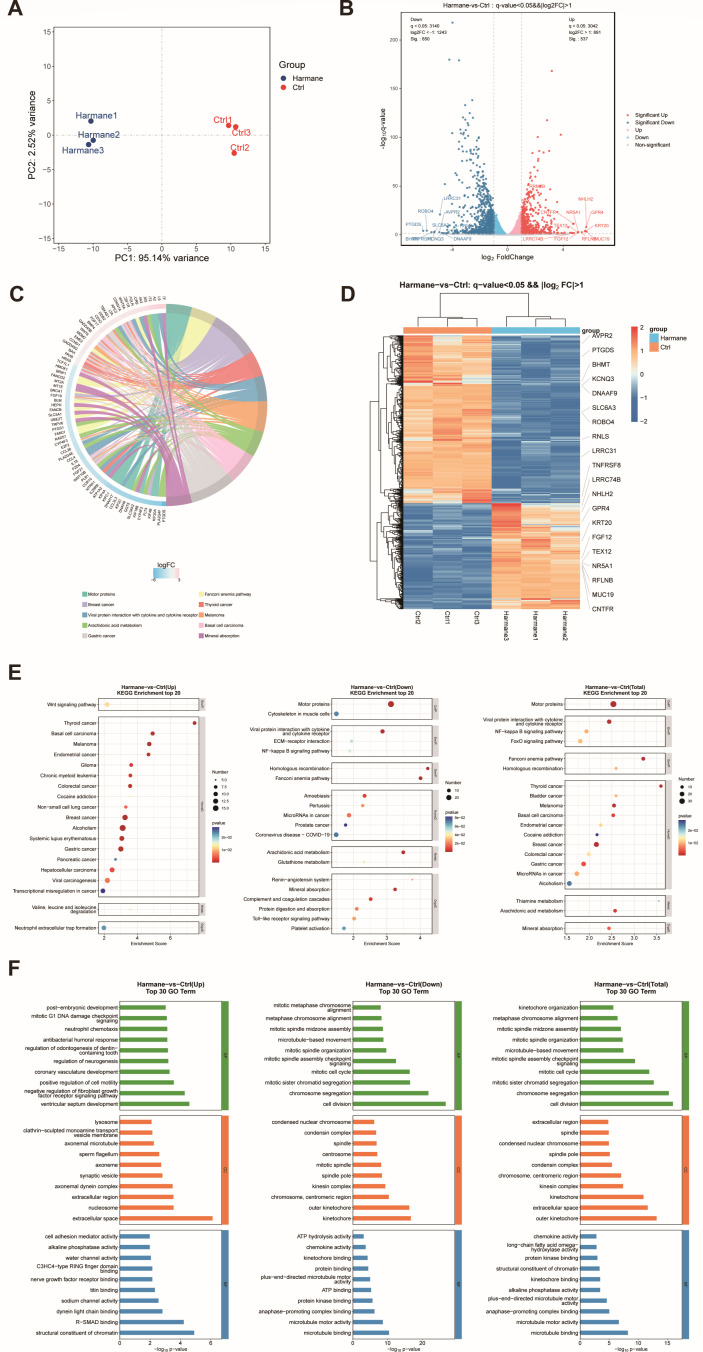
Transcriptomic profiling and pathway analysis reveal molecular mechanisms underlying the anticancer effects of harmane (*n* = 3). (**A**) PCA of RNA-seq data derived from HCT8 cells treated with harmane and untreated controls. (**B**) Volcano plot visualizing DEGs between the control and harmane-treated groups. (**C, D**) Genome-wide distribution and clustering of DEGs illustrated by (**C**) circos plot and (**D**) a heatmap. (**E, F**) Functional enrichment analysis of DEGs using (**E**) KEGG and (**F**) GO databases.

To further investigate the molecular mechanisms underlying the antitumor effects of harmane, RNA-seq analysis was performed on HCT8 cells treated with harmane versus untreated controls. PCA demonstrated clear separation between the two groups, confirming robust transcriptomic alterations induced by harmane and supporting the reliability of the sequencing results (Fig. 3A). A total of 1,387 DEGs were identified under a threshold of |log_2_(fold change)| > 1 and adjusted *q*-value <0.05. Among these, 537 genes were significantly upregulated, and 850 were downregulated in harmane-treated cells (Fig. 3B). The overall expression patterns were visualized via a circos plot and heatmap, highlighting distinct clustering between treatment and control groups (Fig. 3C and D). KEGG pathway enrichment analysis revealed that DEGs were significantly involved in the Wnt signaling pathway and arachidonic acid metabolism, suggesting harmane’s role in modulating key oncogenic and metabolic processes (Fig. 3E). GO enrichment analysis further indicated that DEGs were mainly associated with biological processes, including cell cycle regulation, G1/S phase DNA damage checkpoint signaling, and cell division (Fig. 3F). These transcriptomic findings align with previously observed functional phenotypes, indicating that harmane inhibits proliferation-related pathways and induces DNA damage-mediated cell cycle arrest in CRC cells.

### Harmane induces apoptosis in CRC cells through RRM2B

**Fig 4:**
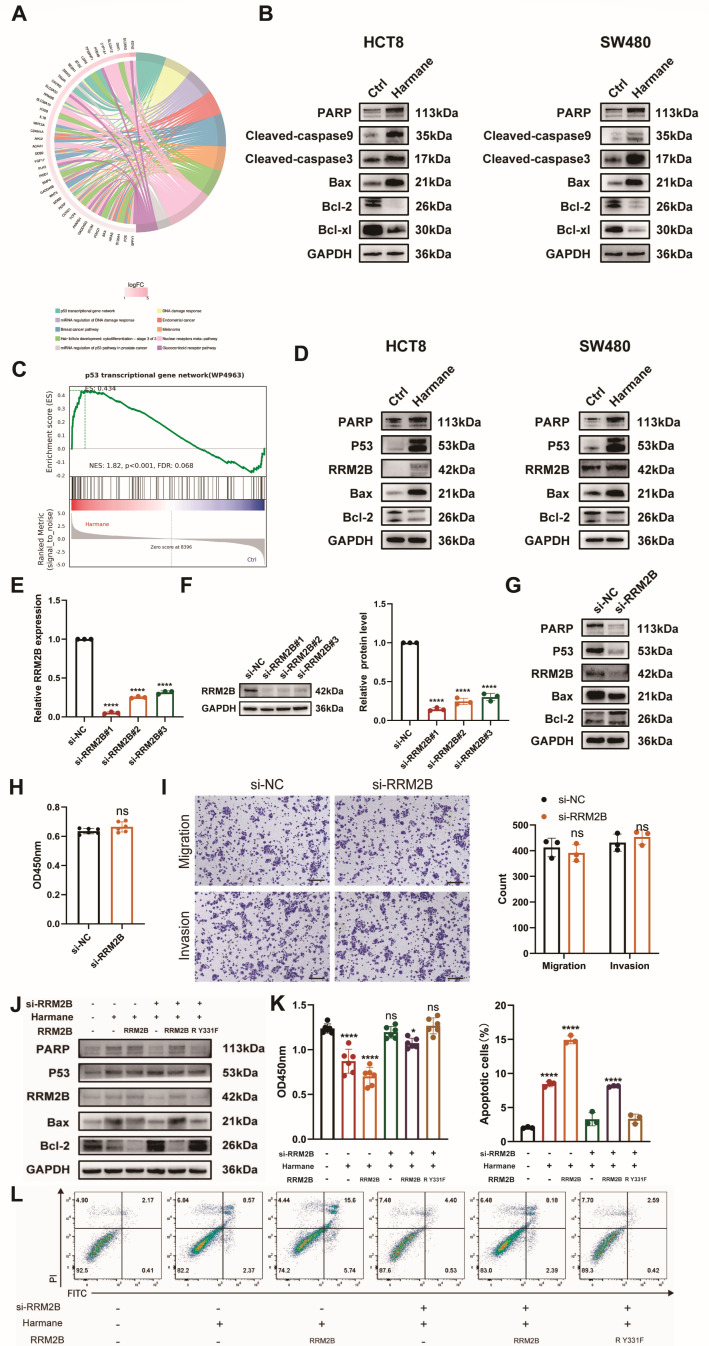
Harmane induces apoptosis and cell cycle arrest in CRC cells through RRM2B. (**A**) Circos plot displaying differential expression patterns of apoptosis-related genes between control and harmane-treated HCT8 cells. (**B**) Western blot analysis of apoptosis-related protein expression in CRC cells after harmane treatment. (**C**) GSEA illustrating significant activation of the p53 signaling pathway in harmane-treated CRC cells. (**D**) Western blot analysis of p53, its downstream target RRM2B, and apoptosis-related proteins in CRC cells after harmane treatment. (**E, F**) Validation of RRM2B knockdown efficiency using three independent siRNAs via (**E**) qPCR and (**F**) Western blot analysis. Statistical significance was determined using one-way analysis of variance (ANOVA). (**G**) Western blot analysis of apoptosis-related proteins following RRM2B knockdown. (**H**) Cell viability was assessed by CCK-8 assay after RRM2B silencing and harmane treatment in CRC cells. Statistical significance was determined using a *t*-test. (**I**) Metastatic capabilities were evaluated by transwell migration and invasion assays after RRM2B silencing and harmane treatment in CRC cells. (**J**) After knockdown of endogenous RRM2B, RRM2B or its catalytically inactive mutant (RRM2B Y331F) was overexpressed to investigate its effects on harmane-induced apoptosis in CRC cells. Statistical significance was determined using two-way analysis of variance (ANOVA). (**K, L**) Cell viability and apoptosis were assessed by (**K**) CCK-8 assay and (**L**) flow cytometry to evaluate the effects of overexpression of RRM2B or its catalytically inactive mutant (RRM2B Y331F) on harmane-induced apoptosis in CRC cells following knockdown of endogenous RRM2B. Statistical significance was determined using two-way analysis of variance (ANOVA). All data were derived from one representative experiment out of three independent biological replicates. Results are presented as means ± SEM. ***P < 0.005, ****P < 0.001.

RNA-seq analysis showed that harmane treatment significantly regulated the expression of apoptosis-related genes, including *Bax, GADD45B*, and *GADD45G* (Fig. 4A). Western blotting analysis further demonstrated that harmane decreased the expression of anti-apoptotic proteins Bcl-2 and Bcl-xL while concurrently enhancing the expression of pro-apoptotic Bax, cleaved-caspase-3, and cleaved caspase-9, indicating activation of the intrinsic apoptotic pathway. Additionally, upregulation of PARP suggested that harmane-induced apoptosis might be mediated through DNA damage mechanisms (Fig. 4B). Moreover, pathway enrichment analysis revealed that harmane treatment affected cell cycle regulation (Fig. S2A). GSEA supported these findings, demonstrating a pronounced arrest in cell cycle progression (Fig. S2B). Consistently, harmane treatment downregulated the expression of several key proteins that promote cell cycle progression, including cyclin E1, cyclin D1, and CDK2, while the expression of the cell cycle inhibitor p21 was markedly upregulated (Fig. S2C). In addition, harmane reduced the expression of critical cell cycle–promoting genes, such as c-Myc and CycD (Fig. S2D), accompanied by a substantial increase in *p53* transcription (Fig. 4C). Among p53 target genes, RRM2B (p53R2) was identified as a potential mediator. Subsequent experiments confirmed that harmane treatment elevated both p53 and RRM2B protein levels (Fig. 4D). To elucidate the functional role of RRM2B in harmane-induced apoptosis, three different siRNAs were designed to knock down RRM2B expression. Based on qPCR and Western blot analyses, sequence one achieved the most efficient knockdown and was selected for further experiments (Fig. 4E and F). SiRNA-mediated depletion of RRM2B notably attenuated harmane’s pro-apoptotic effects: Bcl-2 expression was restored, while expression levels of Bax, p53, RRM2B, and PARP were reduced (Fig. 4G). Functional assays corroborated these findings, demonstrating that RRM2B knockdown reversed harmane-mediated suppression of cell viability, migration, and invasion (Fig. 4H and I). Together, these results indicate that harmane induces apoptosis and cell cycle arrest in CRC cells through RRM2B. Next, we sought to determine whether the pro-apoptotic effect of RRM2B depends on its ribonucleotide reductase activity. Previous studies have identified Y49, Y285, and Y331 as critical residues required for maintaining the enzymatic activity and redox function of RRM2B (24, 25). Therefore, we constructed RRM2B mutant overexpression plasmids (Y49F, Y285F, and Y331F) to evaluate the contribution of these residues to its pro-apoptotic function. Initial screening using CCK-8 assays and Western blot analysis revealed that the Y331F mutant exhibited protein expression levels comparable to the wild type but failed to induce apoptosis (Fig. S2E and F). Based on these findings, the Y331F mutant was selected for subsequent experiments. Further analysis demonstrated that the catalytically inactive RRM2B mutant (Y331F) was unable to induce apoptosis in CRC cells, indicating that the pro-apoptotic function of RRM2B is dependent on its ribonucleotide reductase activity (Fig. 4J, K, and L). Moreover, these results suggest that alterations in dNTP metabolism may contribute to harmane-induced replication stress, thereby activating apoptotic signaling pathways.

### Harmane inhibits tumor growth and induces apoptosis in a CRC xenograft model

**Fig 5:**
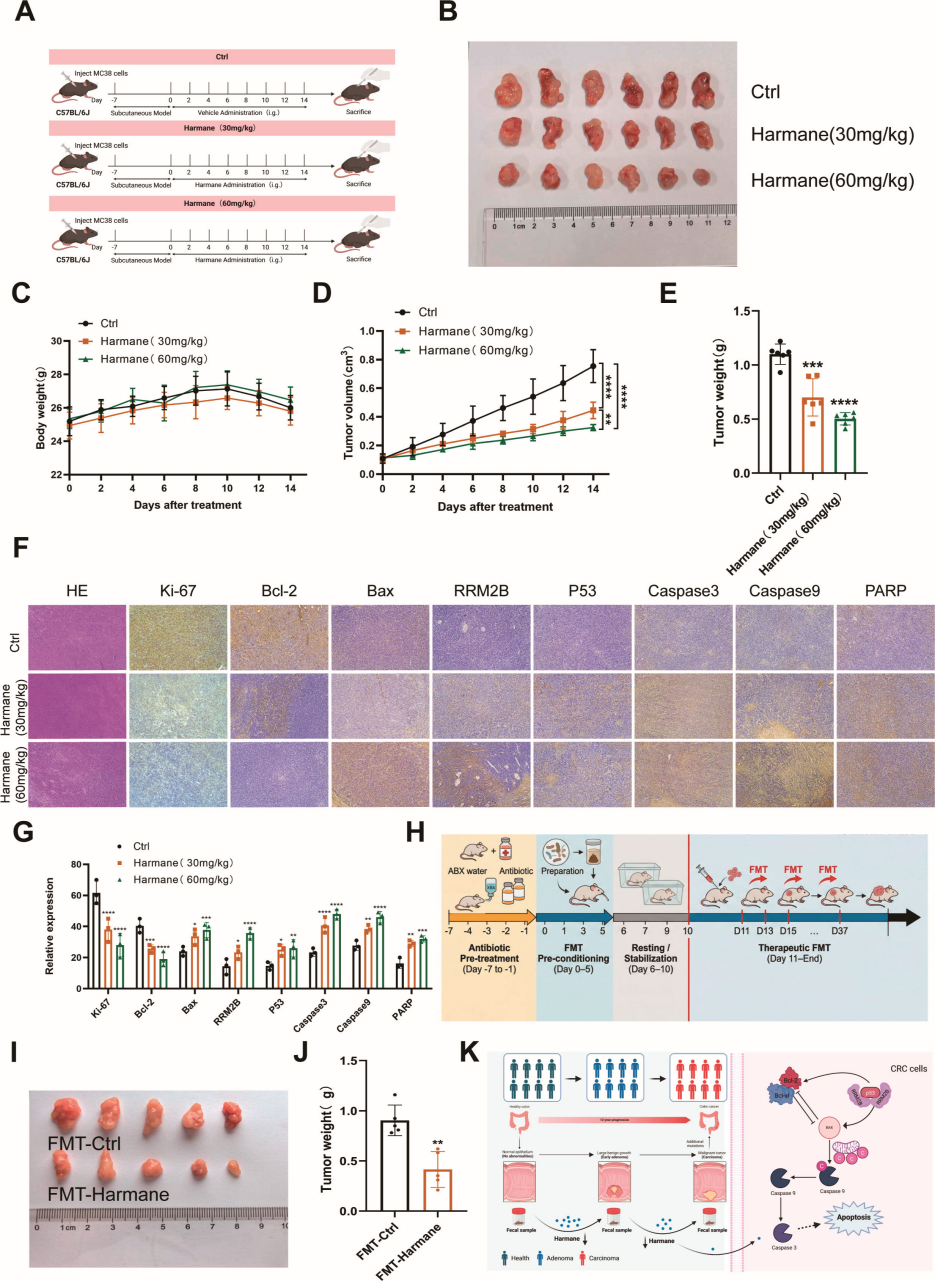
Harmane inhibits CRC progression *in vivo* (*n* = 6 per group). (**A**) Experimental timeline illustrating MC38 tumor cell implantation and harmane administration in C57BL/6J mice. (**B**) Representative images of excised mouse tumors from control and harmane-treated groups. (**C**) Body weight records of mice in the control and harmane-treated groups throughout the experiment. Statistical significance was determined using one-way analysis of variance (ANOVA). (**D, E**) Comparisons of (**D**) tumor volume progression and (**E**) final tumor weight in control and harmane-treated mice. Statistical significance was determined using one-way ANOVA (**F, G**) Immunohistochemical analysis of tumor proliferation and apoptosis between control and harmane-treated mice. Statistical significance was determined using two-way ANOVA (**H**) Schematic diagram of fecal microbiota transplantation (FMT). (**I**) Representative images of mouse tumors from the fecal microbiota transplantation control and the harmane-treated group. (**J**) Final tumor weight of mice in the fecal microbiota transplantation control and harmane-treated groups. Statistical significance was determined using a *t*-test. (**K**) Schematic diagram illustrating that harmane, a differential metabolite identified in the intestines of healthy individuals, advanced adenoma patients, and CRC patients, induces apoptosis in CRC cells through RRM2B-mediated pathways, leading to suppression of tumor growth and progression. Data are derived from one representative experiment out of three independent biological replicates. Results are presented as means ± SEM. *P < 0.05, **P < 0.01, ***P < 0.005, ****P < 0.001.

To evaluate the antitumor efficacy of harmane *in vivo*, an MC38 cell-derived xenograft mouse model was established. The experimental timeline outlining tumor inoculation and harmane administration via oral gavage is detailed in Fig. 5A. Harmane treatment significantly suppressed tumor growth, as evidenced by reduced tumor volume (Fig. 5B and D) and decreased tumor weight at the endpoint (Fig. 5E). Notably, no significant differences in body weight were observed between the control and harmane-treated groups throughout the study (Fig. 5C), indicating minimal systemic toxicity at the administered dosage. To further explore the mechanism underlying harmane-induced tumor suppression, immunohistochemical staining of tumor tissues was performed to assess key markers of proliferation and apoptosis. Harmane-treated tumors exhibited significantly increased apoptosis compared with the control group (Fig. 5F and G). To further establish a causal relationship, we performed FMT) experiments. Mice were first pretreated with a broad-spectrum antibiotic cocktail for 1 week to deplete their endogenous gut microbiota. During CRC model establishment and following previously reported methods, fecal suspensions (200 mg/mL in PBS) prepared from control and harmane-treated donor mice were orally administered (200 µL per mouse every other day) to two groups of recipient mice until the end of the experiment (26). The FMT results showed that the gut microbiota derived from harmane-treated mice significantly attenuated tumor growth (Fig. 5I and J). These *in vivo* findings suggest that harmane may effectively inhibit CRC progression by reshaping the gut microbiota and inducing apoptosis. This is consistent with our *in vitro* results and supports its potential as a therapeutic agent for CRC intervention.

### Harmane alters the intestinal metabolic profile in mice

**Fig 6:**
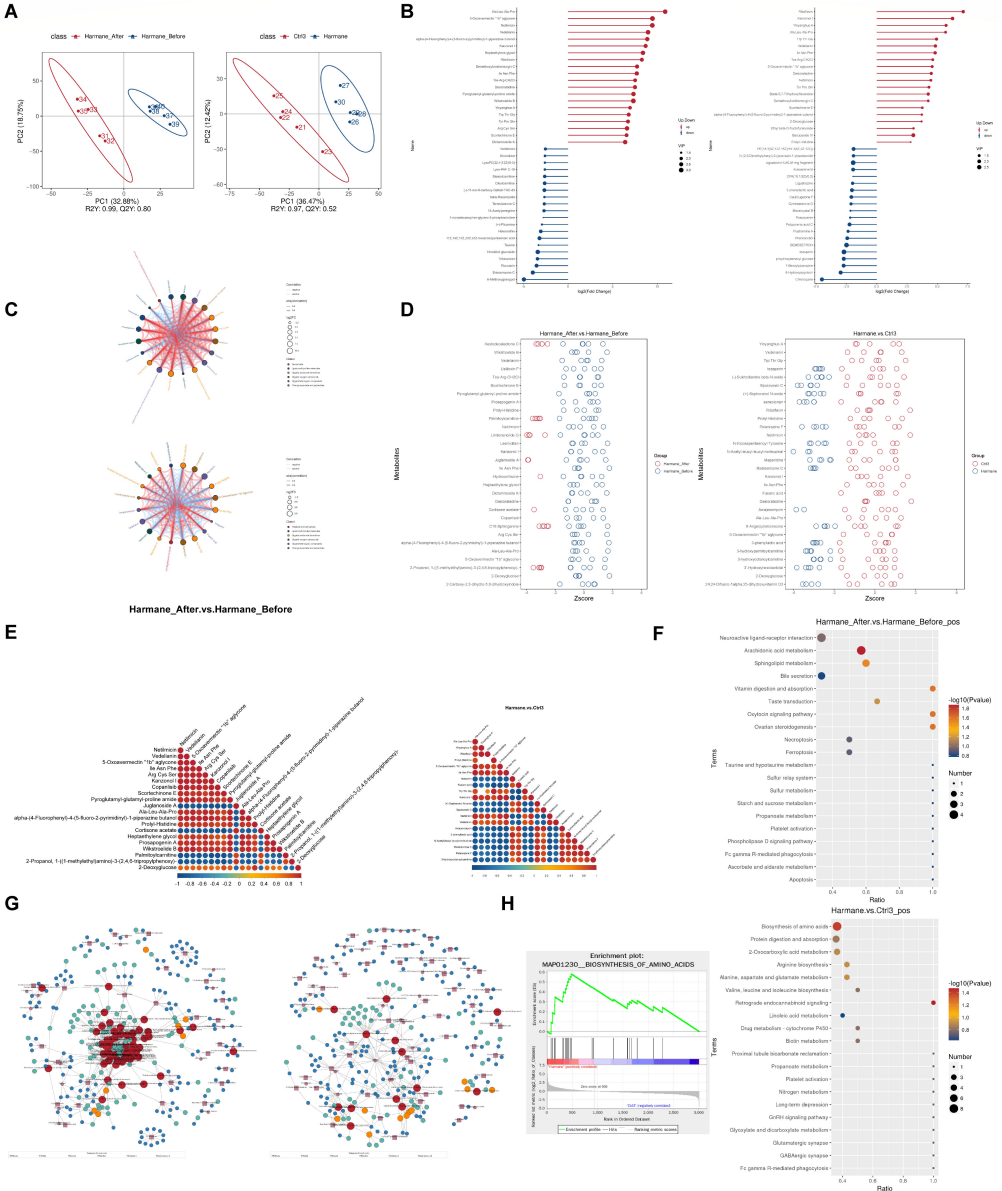
Harmane remodels the gut metabolomic profile in mice. (**A**) PLS-DA of fecal metabolites in C57BL/6J mice between pre-and post-harmane gavage groups, as well as between harmane-treated and untreated groups (positive ion mode). (**B**) Lollipop plot displaying the top 20 significantly upregulated and downregulated metabolites in response to harmane administration. (**C**) Chord diagram illustrating key correlation patterns among differentially abundant metabolites following harmane exposure. (**D**) *z*-score plot showing significantly altered metabolites across experimental groups. (**E**) Correlation analysis of differential metabolites revealing functional associations and potential biochemical interactions influenced by harmane. (**F**) KEGG enrichment analysis of differential metabolites identifying major biological pathways perturbed by harmane treatment. (**G**) KEGG regulatory network diagram illustrating potential mechanistic links between harmane-induced metabolite changes and functional outcomes in the gut microenvironment. (**H**) GSEA confirming significant enrichment of metabolic pathways in harmane-treated mice compared with controls.

Following the observation that harmane suppresses CRC growth in mice and was identified as a differential metabolite in the gut, we investigated its impact on the composition of intestinal metabolites before and after administration. Non-targeted metabolomic analysis of murine feces and cecal contents was performed using LC-MS. PLS-DA revealed distinct clustering of metabolic profiles between pre- and post-harmane administration, indicating significant alterations in the gut metabolome following treatment. Similarly, pronounced separation was observed between harmane-treated and control groups (Fig. 6A; Fig. S3A). Comparative analysis identified multiple metabolites exhibiting concentration changes after gavage. Notably, Ala-Leu-Ala-Pro, 5-oxoavermectin “1b” aglycone, netilmicin, vedelianin, alpha-(4-fluorophenyl)-4-(5-fluoro-2-pyrimidinyl)-1-piperazine, butanol, and kanzonol I were significantly elevated, whereas 6-methoxygossypol, eriosemaone C, taurine, roccanin, trihexanoin, and hinokitiol glucoside were reduced. Compared with controls, harmane-treated mice showed elevated levels of riboflavin, kanzonol I, yinyanghuo A, Ala-Leu-Ala-Pro, and Trp-Thr-Gly, along with decreased concentrations of chloroquine, 1-benzylpiperazine, 8-hydroxyacyclovir, p-hydroxybenzoyl glucose, and teaspenn in the intestinal lumen (Fig. 6B; Fig. S3B and Eand S6E). Chord diagram analysis revealed that, before and after harmane gavage, Ala-Leu-Ala-Pro was positively correlated with prolyl-histidine, and 5-oxoavermectin “1b” aglycone was positively correlated with vedelianin. In the comparison between control and harmane-treated groups, 5-oxoavermectin “1b” aglycone showed a positive association with Ile-Asn-Phe, and kanzonol I was positively correlated with Trp-Thr-Gly, but negatively correlated with fusaric acid (Fig. 6C; Fig. S3C). Z-score analysis further supported the consistency and robustness of these metabolic shifts (Fig. 6D; Fig. S3D). KEGG pathway enrichment analysis indicated that metabolic changes before and after harmane administration were mainly enriched in arachidonic acid metabolism and sphingolipid metabolism. In contrast, differences between harmane-treated and untreated mice were predominantly associated with the biosynthesis of amino acids, protein digestion and absorption, and 2-oxocarboxylic acid metabolism (Fig. 6F; Fig. S3F and G and S6G). GSEA analysis further demonstrated significant upregulation of amino acid biosynthesis pathways in the harmane-treated group compared with controls (Fig. 6H; Fig. S3H). Together, these findings demonstrate that harmane substantially reshapes the metabolic landscape of the mouse gut, with a notable emphasis on reprogramming amino acid metabolism, a process critically implicated in CRC progression.

### Harmane alters the composition and functional potential of the gut microbiota in mice

**Fig 7:**
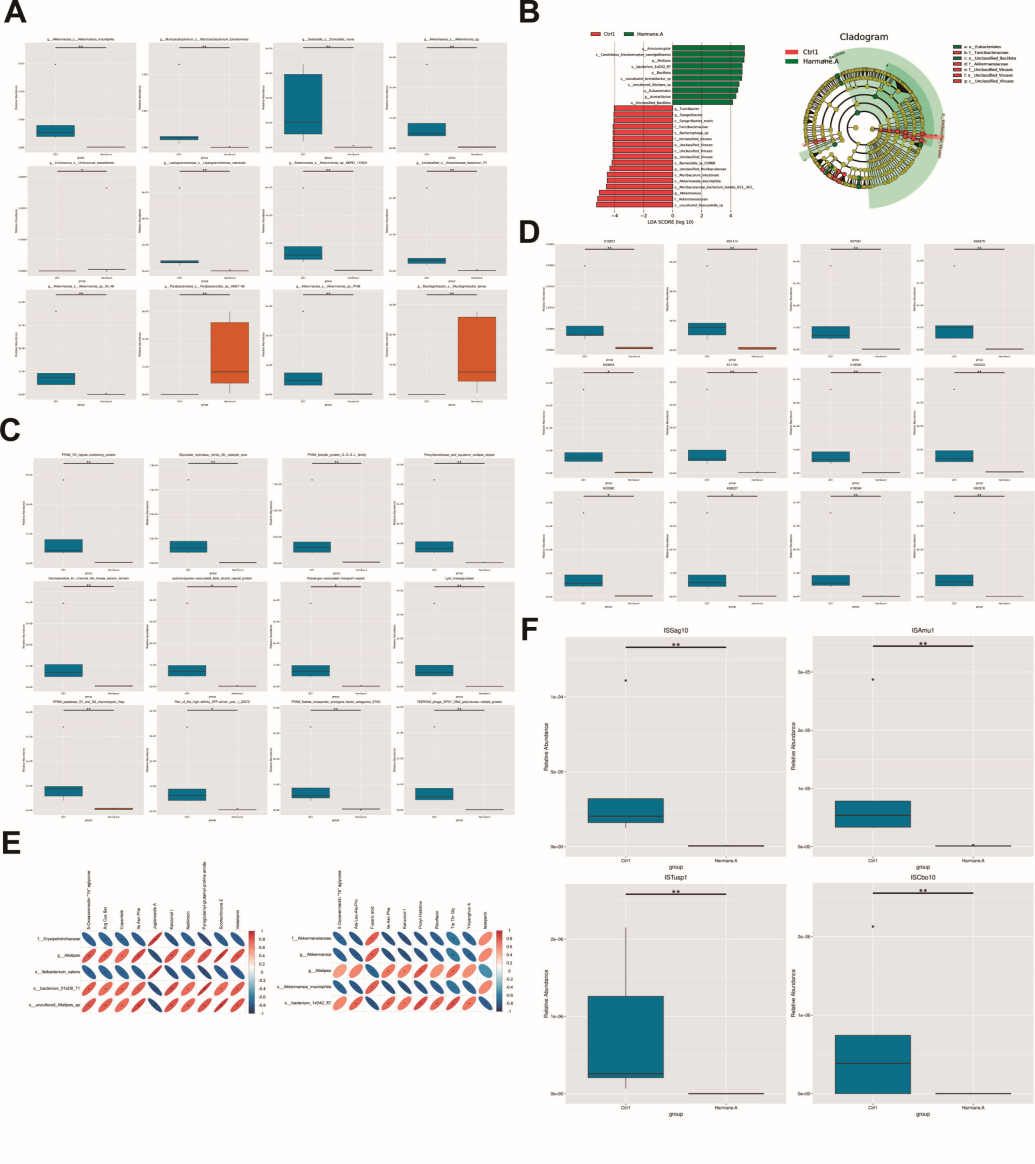
Harmane remodels the gut microbial community structure and functional potential in mice. (**A**) Differentially abundant bacterial taxa analyzed by MetaGenomeSeq between control and harmane-treated mice using fecal samples. (**B**) LDA score distribution and cladogram from LEfSe analysis showing differential enriched microbial taxa between control and harmane-treated groups. (**C, D**) Functional annotation of metagenomic data using (**C**) eggNOG and (**D**) KEGG. (**E**) Abundance changes of MGEs following harmane treatment. (**F**) Integration analysis of metagenomic and metabolomic data, displaying Spearman correlation coefficients between significantly altered microbial species and intestinal metabolites across control and harmane-treated groups.

Metagenomic analysis of fecal samples was conducted to determine whether harmane-induced metabolic changes in the gut were associated with structural and functional alterations in the microbiota. Harmane administration significantly shifted microbial community composition, characterized by a decrease in the abundance of *Muricaebacterium torontonense*, *Dubosiella muris*, *Akkermansia muciniphila*, and *Leptogranulimonas caecicola*, while promoting the growth of *Parabacteroides* sp. AM27-42 and *Mucilaginibacter terrae* (Fig. 7A). LEfSe analysis, including LDA bar plots and cladograms, further revealed significant enrichment of the genera *Amulumruptor* and *Alistipes*, along with the phylum *Bacillota*, in the harmane-treated group compared to controls (Fig. 7B). Consistent with prior functional studies, harmane exposure led to enrichment in cell cycle-related pathways, as illustrated in the LDA value distribution map (Fig. S4A). MetaGenomeSeq analysis demonstrated that harmane downregulated multiple functional categories. At the eggNOG level, reductions were observed in proteins associated with bacterial adhesion and structural maintenance, such as PFAM_YD repeat-containing protein and autotransporter-associated beta-strand repeat proteins (Fig. 7C). Similarly, at the MGE level, harmane treatment suppressed genes encoding autotransporter adhesins, glycoside hydrolases, IS family transposases, and tyrosine recombinases (Fig. 7D), indicating diminished bacterial pathogenicity, antibiotic resistance dissemination, and metabolic adaptability. Additional metagenomic analysis at the CARD and CAZy levels indicated reduced abundance of the *InuC* gene and glycoside hydrolase family 177 (GH177) in harmane-treated mice (Fig. S4B and C). Furthermore, ISfinder analysis confirmed a decline in insertion sequence elements such as ISSag10, ISAmu1, ISTusp1, and ISCbo10 (Fig. 7E), supporting an overall reduction in horizontal gene transfer and antibiotic resistance potential. Integrated correlation analysis between metabolomic and metagenomic data revealed significant associations between specific microbial taxa and metabolites. Pre- and post-harmane treatment, *g_Alistipes* was positively correlated with 5-oxoavermectin “1b” aglycone, Arg-Cys-Ser, copanlisib, and kanzonol I, while *f_Erysipelotrichaceae* exhibited negative correlations with these metabolites. In harmane-treated mice, *f_Akkermansiaceae* was negatively correlated with 5-oxoavermectin “1b” aglycone, Ala-Leu-Ala-Pro, Ile-Asn-Phe, and kanzonol I (Fig. 7F). Collectively, these results demonstrate that harmane remodels the gut microbial ecosystem by altering its composition, reducing pathogenic features, suppressing antibiotic resistance mechanisms, and modulating metabolite-microbe interactions, ultimately contributing to the inhibition of CRC progression.

## DISCUSSION

CRC is one of the most common malignant tumors worldwide. Although multiple therapeutic strategies are currently available, chemotherapy remains the primary treatment, and the development of chemoresistance often limits its clinical efficacy. Harmane is a β-carboline alkaloid naturally present in plants, foods, and mammalian tissues. Previous studies have shown that harmane possesses antitumor activity; however, its mechanism of action in CRC remains unclear. In this study, we demonstrate that harmane suppresses CRC progression through dual mechanisms involving RRM2B-mediated cell cycle arrest and apoptosis, as well as modulation of the gut microbiota. At the cellular level, harmane inhibited CRC cell proliferation by inducing G0/G1 phase cell cycle arrest and apoptosis. It has been reported that the β-carboline derivative B-9-3 exhibits anticancer activity *in vitro* by inducing apoptosis and inhibiting cell migration (9), while another novel harmane dimer, B-9-8, can reverse ABCG2-mediated chemoresistance (27). Moreover, harmane has been identified as a topoisomerase I inhibitor with anticancer potential (28). Previous studies have mainly focused on harmine, the methoxylated derivative of harmane, and have confirmed its anticancer activity in various tumor types. For example, harmine induces pro-death autophagy and apoptosis in human gastric cancer cells (29); causes breast cancer cells to arrest at the G2/M phase via regulation of MAPKs and the AKT/FOXO3a signaling pathways (30); and induces autophagy and apoptosis in osteosarcoma cells via the ROS/p38 axis (31). In this study, we found that harmane slowed cell cycle progression and arrested CRC cells at the G0/G1 phase. This is consistent with previous reports that the β-carboline scaffold of harmane interacts with tumor cell DNA and induces double-strand breaks. Western blot analysis showed a marked increase in PARP, further confirming harmane-induced DNA damage and the suppression of CRC proliferation. DNA damage stabilized and activated p53, which subsequently induced RRM2B expression to coordinate nucleotide metabolism and DNA repair under stress conditions. Under severe damage, upregulated RRM2B, together with the p53–Bax axis, tipped the balance towards mitochondrial apoptosis, leading to cytochrome c release, caspase-9/3 cascade activation, and ultimately cell apoptosis.

Our study also revealed an important role of the gut microbiota in the antitumor effects of harmane. Metagenomic analysis showed that harmane treatment significantly reshaped the gut microbial composition, with increased abundance of taxa such as *Alistipes*, *Amulumruptor*, and *Acetatifactor*, which are associated with amino acid metabolism, carbohydrate degradation, and short-chain fatty acid production. In contrast, taxa associated with inflammation or microbial dysbiosis, such as *Turicibacter* and *Muribaculaceae*, were relatively reduced. The decrease in *A. muciniphila* appears counterintuitive, as this species has been widely associated with beneficial effects in cancer, particularly in enhancing responses to immunotherapy. However, its role in CRC is likely context-dependent and influenced by the overall microbial ecosystem and treatment conditions. In our study, harmane induced a global restructuring of the gut microbiota rather than modulating a single bacterial species. Functional metagenomic analysis further indicated that genes related to bacterial motility, ABC transport systems, and DNA replication were decreased after harmane treatment, suggesting a suppression of microbial functions associated with pathogenicity and rapid proliferation. A growing body of evidence indicates that the gut microbiota plays an indispensable role in CRC progression (32–34). For example, gut microbiota from CRC patients has been shown to promote the development of intestinal adenomas in Apc^min/+^ mice (35); alterations in the gut microbiota of obese patients facilitate colorectal carcinogenesis in mice (36); and appendectomy-induced enrichment of *Bacteroides*, *Veillonella*, and *Prevotella*, along with depletion of beneficial commensals (Blautia YL58, Enterococcus hirae, and Family XII AD3011 group), promotes CRC occurrence (37). In addition, Gasdermin D has been reported to promote intestinal tumorigenesis by regulating IL-1β release and shaping gut microbial composition (38), whereas YYFZBJS alleviates CRC progression in Apc^min/+^ mice by remodeling the gut microbiota and suppressing regulatory T-cell generation (39). According to the literature, gut microbiota can exert pro- or anti-tumor effects through both bacterial properties and their metabolites (40, 41). To our knowledge, current reports specifically addressing the interaction between harmane (or its analogs) and gut microbiota are very limited. This study is among the first to link harmane exposure with structural and functional changes in the gut microbiota, highlighting its dual mechanisms of action—direct tumor cell cytotoxicity and ecological modulation of the gut microbiota.

Consistent with the microbial changes, metabolomic analysis revealed significant alterations in fecal metabolite profiles, including increased levels of amino acid-related metabolites such as Arg-Cys-Ser and several microbiota-derived metabolites. These metabolic changes suggest that harmane may reshape the intestinal metabolic environment through microbiota-mediated metabolic pathways. Importantly, fecal microbiota transplantation experiments further confirmed the functional role of harmane-modulated microbiota. Transplantation of fecal microbiota from harmane-treated mice into antibiotic-treated recipient mice resulted in significantly smaller tumor volumes compared with mice receiving control microbiota. These results indicate that harmane-induced alterations in gut microbiota contribute to its antitumor effects.

Despite these findings, our study has several limitations. For instance, harmine in combination with the PARP inhibitor olaparib has shown efficacy in BRCA1/2 wild-type ovarian cancer (42); harmine hydrochloride induces G2 phase arrest and apoptosis in MGC-803 and SMMC-7721 cells through upregulation of p21, activation of the caspase-8/Bid pathway, and downregulation of the ERK/Bad pathway (30); and harmine derivatives have been designed and evaluated as topoisomerase I inhibitors for cancer therapy (43). In these mechanistic studies, autophagy-related experiments often used inhibitors such as 3-MA (early stage), Bafilomycin A1, and CQ/HCQ, whereas apoptosis-related experiments commonly employed Z-VAD-FMK and Q-VD-OPh. In contrast, our study did not use molecular inhibitors for intervention, which limits our ability to establish causal inferences. The concentrations used *in vitro* may not directly reflect achievable levels *in vivo*, and further pharmacokinetic studies are required to determine clinically relevant dosing. Finally, since RRM2B is a well-established downstream target of p53, the functional effects of harmane may be influenced by the p53 status in CRC. The precise role of p53 in harmane-induced RRM2B activation and the regulation of CRC cell fate remains to be systematically investigated. Due to the scope of the current study, we have not performed comprehensive comparisons using isogenic models with defined p53 status. Future studies will focus on utilizing genetically defined models to further dissect the relative contributions of p53-dependent and p53-independent mechanisms in mediating the anti-tumor effects of harmane.

In conclusion, our study demonstrates that harmane exerts anti-tumor effects through both tumor-intrinsic mechanisms and microbiota-dependent pathways. Harmane induces cell cycle arrest and apoptosis via the p53–RRM2B axis, and its pro-apoptotic effect depends on the ribonucleotide reductase activity of RRM2B. Furthermore, fecal microbiota transplantation experiments establish the potential role of gut microbiota remodeling in mediating harmane-induced suppression of CRC progression.

## Data Availability

All data are available upon reasonable request. The raw metagenomic sequencing data have been deposited in the NCBI BioProject database under accession number PRJNA1313833, and the raw transcriptomic sequencing data have been deposited under accession number PRJNA1313752. The differential metabolites identified from the untargeted metabolomics analysis of mouse gut microbiota are provided in Data Set S1.

## SUPPLEMENTAL MATERIAL

Data Set S1 msystems.01704-25-s0001.xlsx Differential metabolites identified from the untargeted metabolomics analysis of mouse gut microbiota.

Supplemental Figures msystems.01704-25-s0002.docx Figures S1 to S4.

Blots msystems.01704-25-s0003.docx Blot images for Fig. 4B, D, and J and Fig. S2C and F.

Table S1 msystems.01704-25-s0004.docx Reagents.

Table S2 msystems.01704-25-s0005.docx Primers for PCR and real-time PCR.

ASM does not own the copyrights to Supplemental Material that may be linked to, or accessed through, an article. The authors have granted ASM a non-exclusive, world-wide license to publish the Supplemental Material files. Please contact the corresponding author directly for reuse.
