# Supplementary material for: Harmane induces apoptosis through RRM2B and suppresses colorectal cancer progression
Source: mSystems. 2026 Jun 9;11(7):e01704-25. doi: 10.1128/msystems.01704-25 (PMC13386993; doi:10.1128/msystems.01704-25)
Supplement: Supplemental Figures — Figures S1 to S4. [file msystems.01704-25-s0002.docx]

**FigureS1**

**
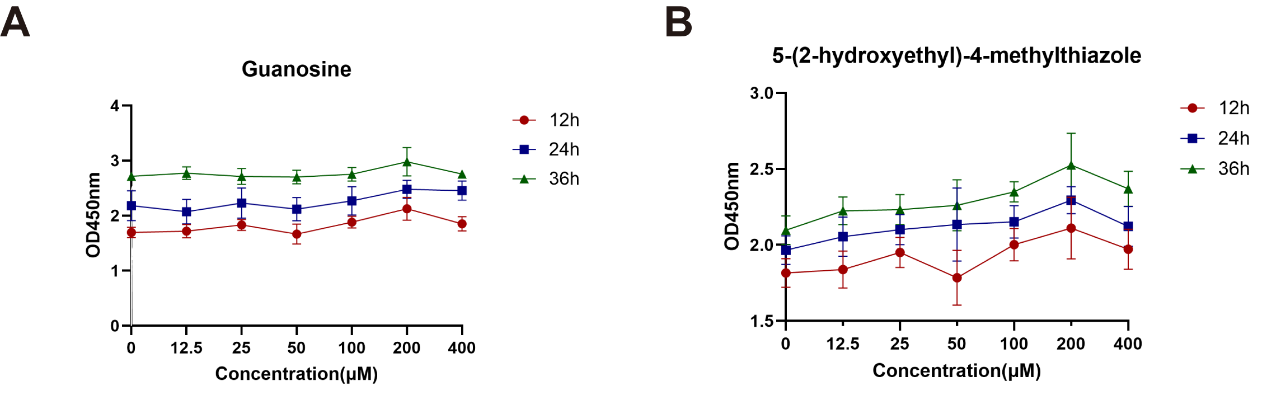
**

**Fig.S1.** During the development and progression of colorectal cancer, the types and levels of metabolites in the human gut undergo changes. (**A**) CCK-8 assay to evaluate the effect of guanosine on CRC cell viability. (**B**) CCK-8 assay to evaluate the effect of 5-(2-hydroxyethyl)-4-methylthiazole on CRC cell viability.

**FigureS2**


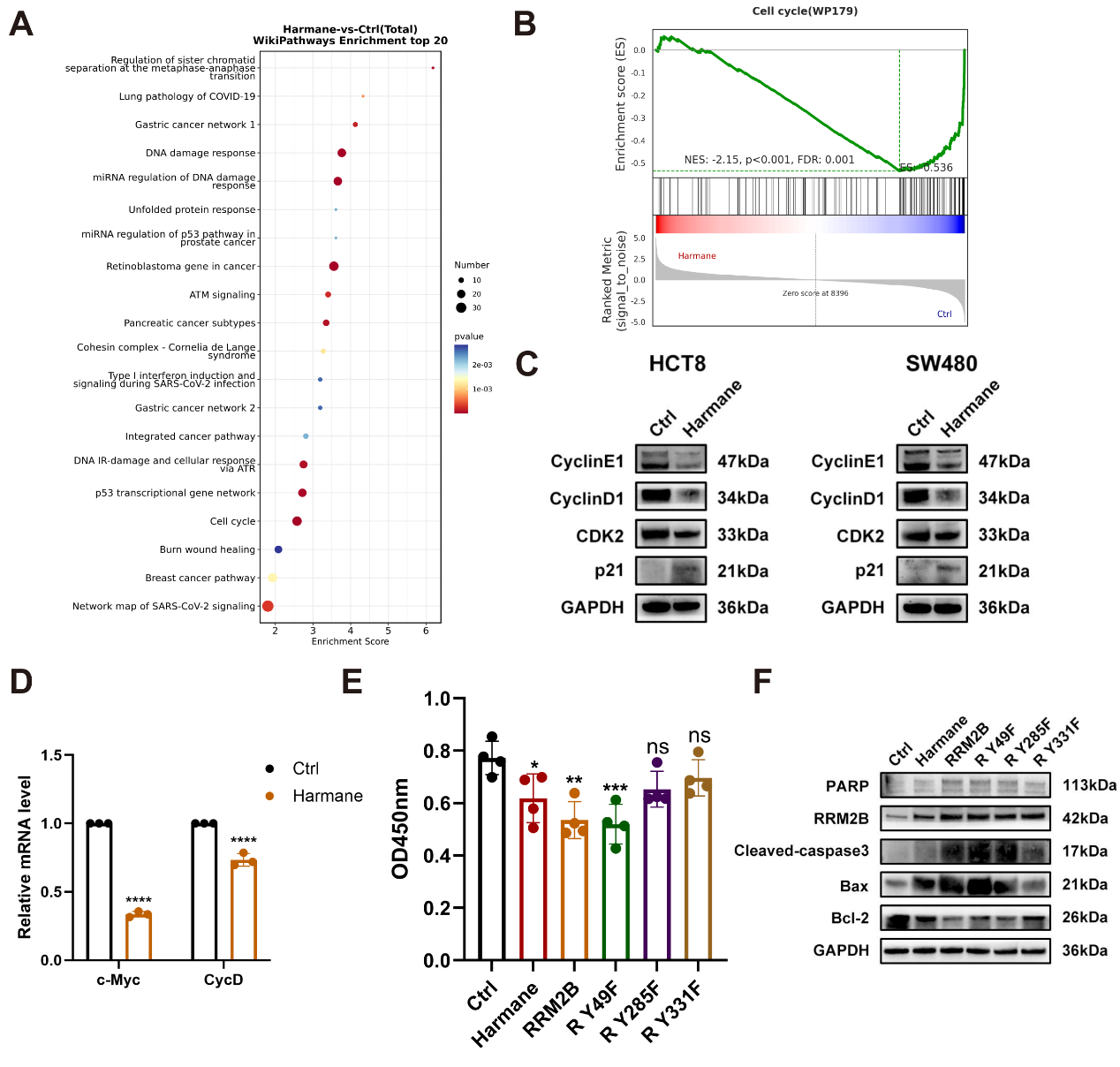


**Fig.S2.** Harmane induces apoptosis and cell cycle arrest in CRC cells through RRM2B. (**A**) Wiki pathway enrichment analyses were performed on differentially expressed genes (DEGs) distinguishing the two groups. (**B**) GSEA reveals the activation status of cell cycle in CRC cells after harmane treatment. (**C**) The expression levels of several key cell cycle–related proteins, including Cyclin E1, Cyclin D1, CDK2, and p21, following harmane treatment. Statistical significance was determined using a t-test. (**D**) MRNA levels of c-Myc and CycD in CRC cells following harmane treatment. Statistical significance was determined using one-way analysis of variance (ANOVA). (**E**)The effects of RRM2B and its site-directed mutants on harmane-induced cytotoxicity were assessed by CCK-8 assay. Statistical significance was determined using one-way analysis of variance (ANOVA). (**F**) The effects of RRM2B and its site-directed mutants on the expression of apoptosis-related proteins induced by harmane. Data were from one representative of three independent experiments. The results are presented as mean ± SEM. **p* < 0.05, ***p* < 0.01, ****p* < 0.005, *****p < 0.001*.

**FigureS3**


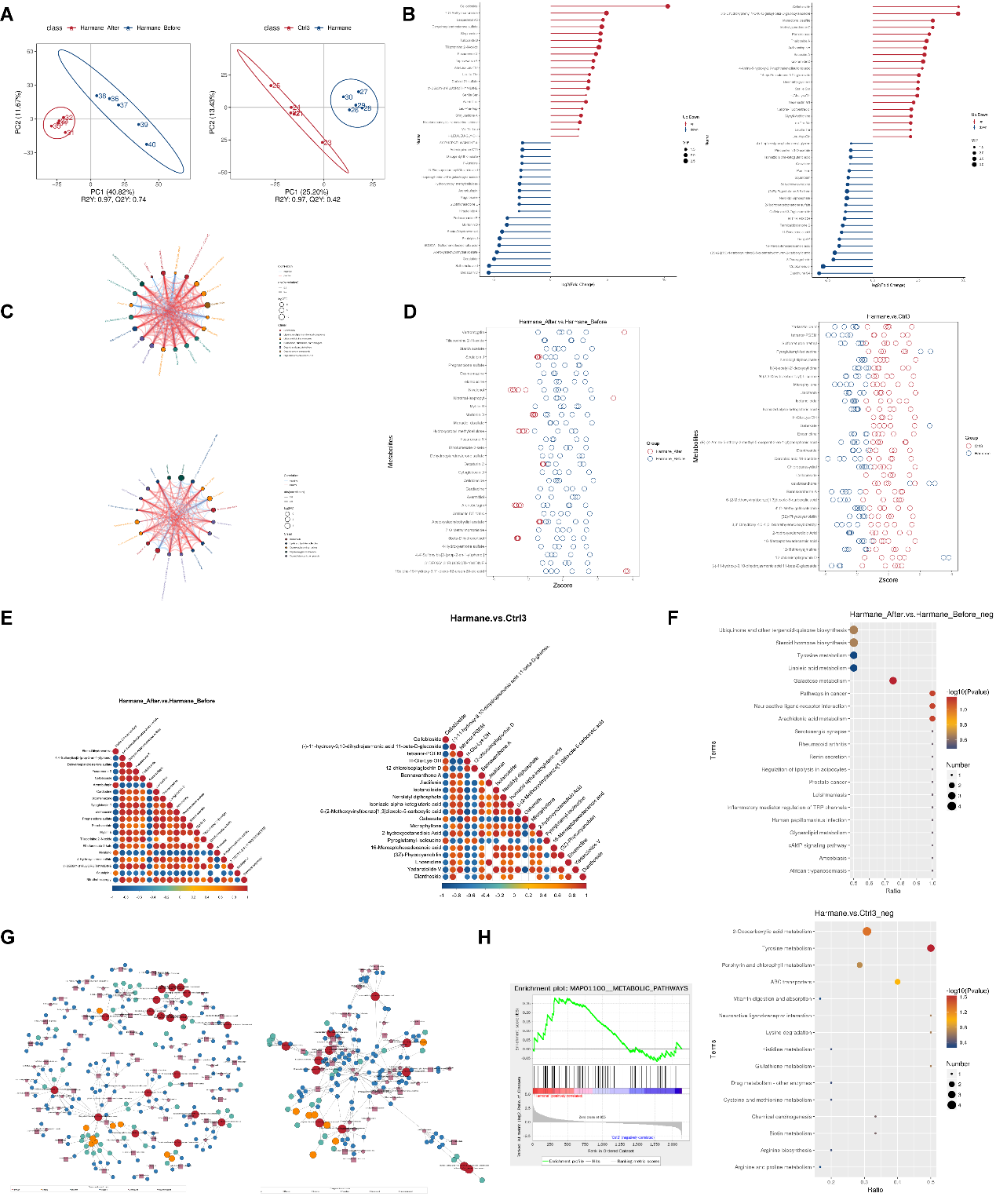


**Fig.S3.** Harmane treatment alters metabolites in the mouse gut. (**A**) PLS-DA shows distinct clustering of gut metabolites in mice before and after Harmane gavage, as well as between Harmane-treated and untreated groups in negative ion mode. (**B**) The differential metabolite lollipop plot shows the top 20 upregulated and downregulated metabolites in mice before and after Harmane gavage, as well as between Harmane-treated and untreated groups in negative ion mode. (**C**) The differential metabolite chord diagram illustrates the correlations and associations among key metabolites before and after Harmane gavage, as well as between Harmane-treated and untreated groups in negative ion mode. (**D**) The Z-score plot shows the upregulated and downregulated key metabolites before and after Harmane gavage, as well as between Harmane-treated and untreated groups in negative ion mode. (**E**) Correlation analysis of differential metabolites reveals the relationships among metabolites before and after Harmane gavage, as well as between Harmane-treated and untreated groups in negative ion mode. (**F**) KEGG enrichment analysis of differential metabolites identifies the related pathways before and after Harmane gavage, as well as between Harmane-treated and untreated groups in negative ion mode. (**G**) KEGG regulatory network diagram illustrating the regulatory relationships of differential metabolites before and after Harmane gavage, as well as between Harmane-treated and untreated groups in negative ion mode. (**H**) GSEA showing the metabolic pathways enriched by differential metabolites between the control and Harmane-treated groups in negative ion mode.

**FigureS4**

**
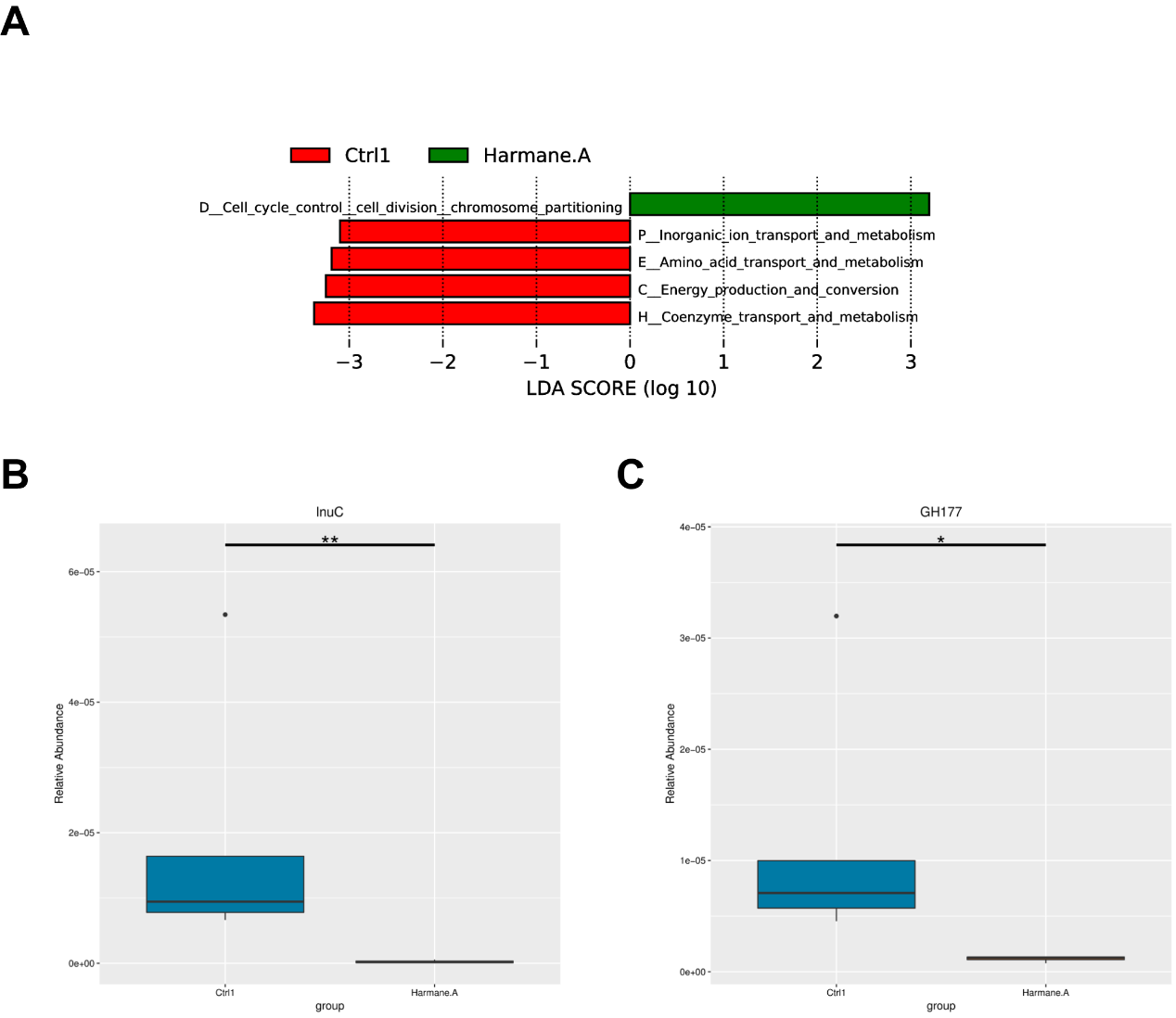
**

**Fig.S4.** Harmane treatment alters the gut microbiota in mice. (**A**) Distribution map of LDA values for differential functions between the harmane treatment group and the control group (**B**) MetaGenomeSeq analysis of faecal samples from harmane-treated and control mice based on CARD levels. (**C**) MetaGenomeSeq analysis of faecal samples from harmane-treated and control mice based on CAZy levels.
