# Supplementary material for: Harmane induces apoptosis through RRM2B and suppresses colorectal cancer progression
Source: mSystems. 2026 Jun 9;11(7):e01704-25. doi: 10.1128/msystems.01704-25 (PMC13386993; doi:10.1128/msystems.01704-25)
Supplement: Blots — Blot images for Fig. 4B, D, and J and Fig. S2C and F. [file msystems.01704-25-s0003.docx]

**Fig.4.B**

**HCT8(Ctrl Harmane) SW480(Ctrl Harmane)**

**PARP


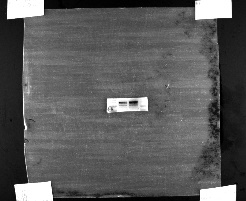



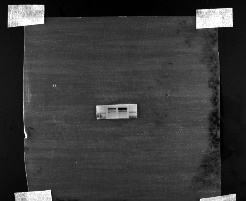
**

**Cleaved-caspase9


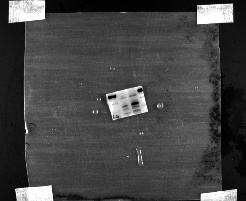



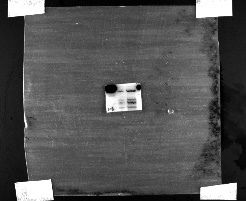
**

**Cleaved-caspase3


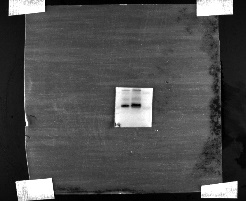



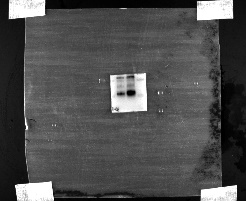
**

**Bax


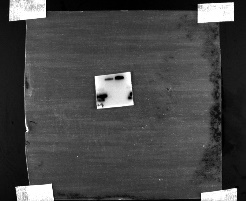



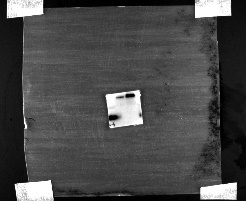
**

**Bcl-2


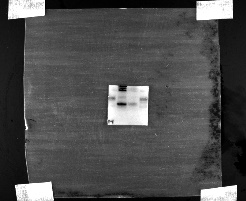



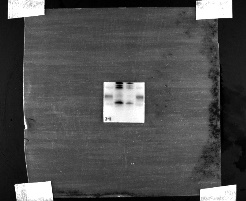
**

**Bcl-xl


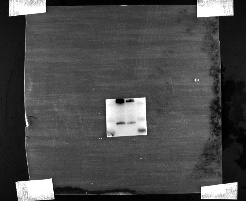



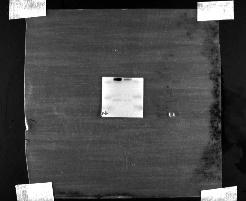
**

**GAPDH**


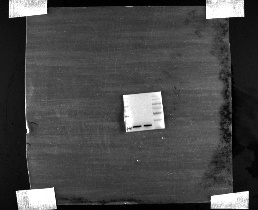
 **


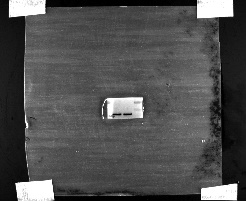
**

**Fig.4.D**

**HCT8(Ctrl Harmane) SW480(Ctrl Harmane)**

**PARP


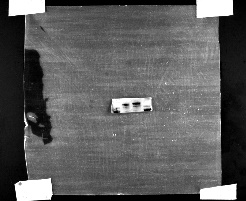



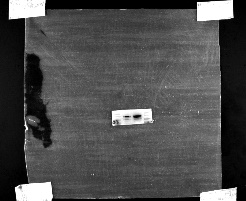
**

**P53


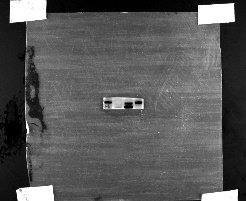



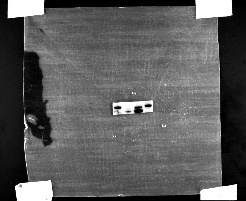
**

**RRM2B


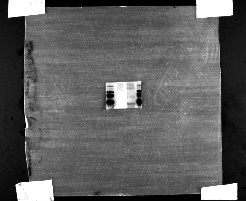



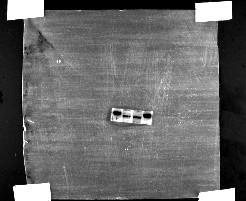
**

**Bax


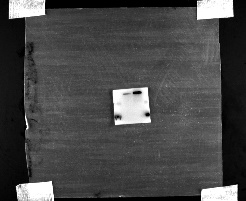



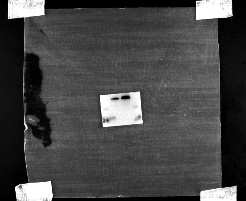
**

**Bcl-2


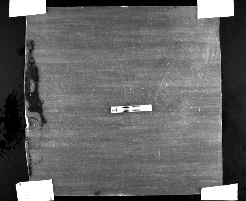



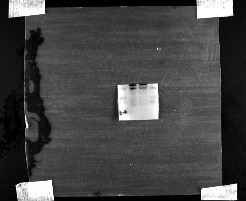
**

**GAPDH


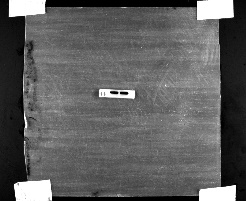



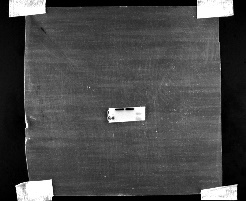
**

**Fig.4.F (si-NC si-RRM2B#1 si-RRM2B#2 si-RRM2B#3)**

**RRM2B


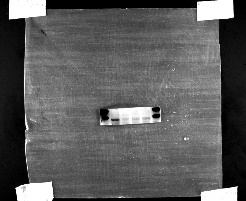
**

**GAPDH


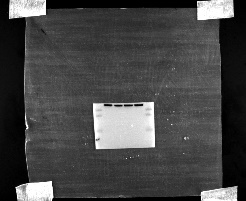
**

**Fig.4.G**

**(si-NC si-RRM2B)**

**PARP


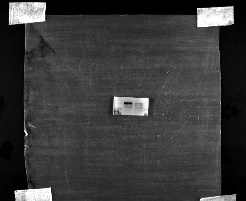
**

**P53


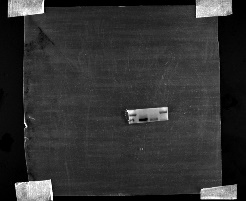
**

**RRM2B


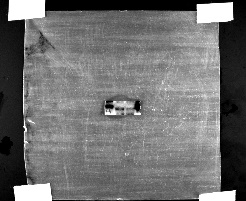
**

**Bax


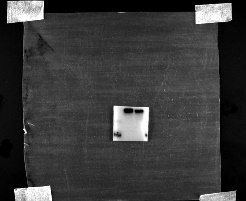
**

**Bcl-2


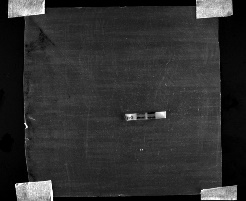
**

**GAPDH


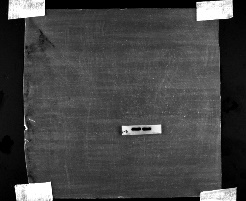
**

**Fig.4.J**

**PARP


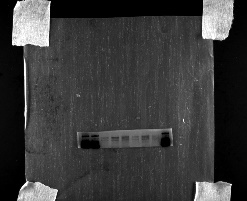
**

**P53


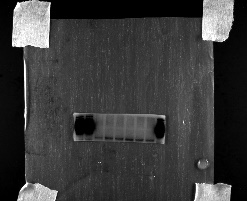
**

**RRM2B


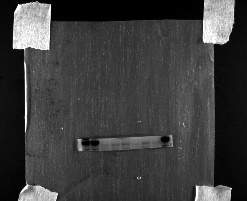
**

**Bax


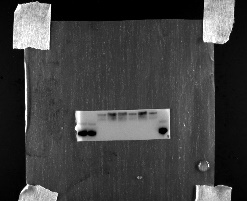
**

**Bcl-2


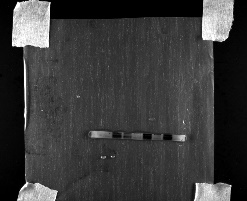
**

**GAPDH


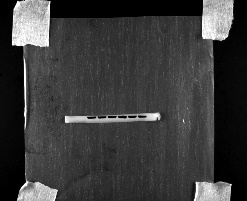
**

**Fig.S2.C**

**HCT8 SW480**

**CyclinE1


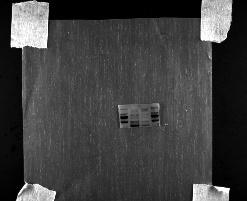



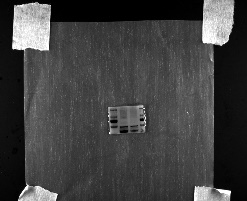
**

**CyclinD1


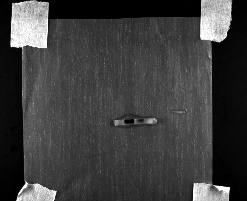



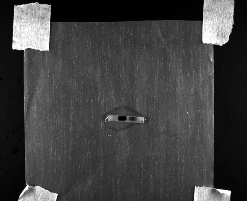
**

**CDK2


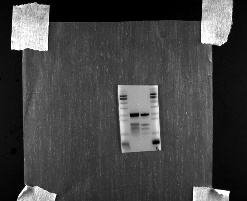



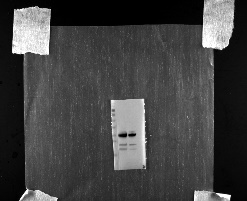
**

**P21


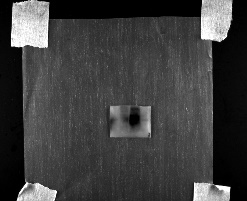



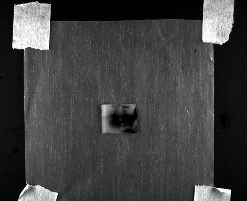
**

**GAPDH


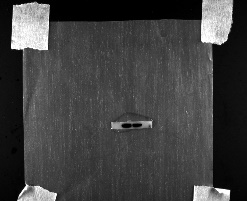



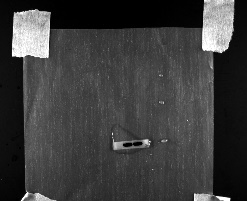
**

**Fig.S2.F**

**PARP**

**RRM2B**

**Cleaved-caspase3**

**Bax**

**Bcl-2**

**GAPDH**
