# Supplementary material for: Harmane induces apoptosis through RRM2B and suppresses colorectal cancer progression
Source: mSystems. 2026 Jun 9;11(7):e01704-25. doi: 10.1128/msystems.01704-25 (PMC13386993; doi:10.1128/msystems.01704-25)
Supplement: Table S1 — Reagents. [file msystems.01704-25-s0004.docx]

**Supplementary Table1**

| **Reagents** | | |
| --- | --- | --- |
| **Reagent** | **Manufacturer** | **Number of catalogues** |
| Anti-67 | Cell Signaling Technology | D3B5 |
| Anti- Bcl-2 | Proteintech | 12789-1-AP |
| Anti- Bcl-xl | Proteintech | 10783-1-AP |
| Anti- Bax | Proteintech | 50599-2-Ig |
| Anti- RRM2B | Proteintech | 18005-1-AP |
| Anti- P53 | Proteintech | 10442-1-AP |
| Anti- Caspase3 | Proteintech | 19677-1-AP |
| Anti- Caspase9 | Proteintech | 10380-1-AP |
| Anti- PARP | Proteintech | 13371-1-AP |
| Anti- GAPDH | Proteintech | 60004-1-Ig |
