# Supplementary material for: Harmane induces apoptosis through RRM2B and suppresses colorectal cancer progression
Source: mSystems. 2026 Jun 9;11(7):e01704-25. doi: 10.1128/msystems.01704-25 (PMC13386993; doi:10.1128/msystems.01704-25)
Supplement: Table S2 — Primers for PCR and real-time PCR. [file msystems.01704-25-s0005.docx]

**Supplementary Table2**

| **Primers for PCR and real time PCR** | | |
| --- | --- | --- |
| **Primers** | **Sequences-F** | **Sequences-R** |
| Human-c-Myc | 5′ -GCAATGCGTTGCTGGGTTAT -3′ | 5′ -TCCCTCCGTTCTTTTTCCCG-3′ |
| Human-CycD | 5′-GCTGCGAAGTGGAAACCAGA-3′ | 5′-CCTCCTTCTGCACACATTTGA-3**′** |
| Human-RRM2B | 5′-GCAGGACCGCTGTAGGAAAT-3′ | 5′-ATGACAAACCGGCGAGAACT-3**′** |
| Human-GAPDH | 5′-AATGGGCAGCCGTTAGGAAA-3′ | 5′ -GCGCCCAATACGACCAAATC-3′ |
| **Sequences for siRNAs** | | |
| si-RRM2B#1 | GGUCAGGGAGAUCAUUGUUTT | AACAAUGAUCUCCCUGACCTT |
| si-RRM2B#2 | CCAGUGAUGGAAUUGUAAATT | UUUACAAUUCCAUCACUGGTT |
| si-RRM2B #3 | GCUAAAGAAGAGAGGUCUUTT | AAGACCUCUCUUCUUUAGCTT |
